# Publisher Correction: Combating subclonal evolution of resistant cancer phenotypes

**DOI:** 10.1038/s41467-017-02383-6

**Published:** 2018-02-05

**Authors:** Samuel W. Brady, Jasmine A. McQuerry, Yi Qiao, Stephen R. Piccolo, Gajendra Shrestha, David F. Jenkins, Ryan M. Layer, Brent S. Pedersen, Ryan H. Miller, Amanda Esch, Sara R. Selitsky, Joel S. Parker, Layla A. Anderson, Brian K. Dalley, Rachel E. Factor, Chakravarthy B. Reddy, Jonathan P. Boltax, Dean Y. Li, Philip J. Moos, Joe W. Gray, Laura M. Heiser, Saundra S. Buys, Adam L. Cohen, W. Evan Johnson, Aaron R. Quinlan, Gabor Marth, Theresa L. Werner, Andrea H. Bild

**Affiliations:** 1 Department of Pharmacology and Toxicology, College of Pharmacy, University of Utah, 30 South 2000 East, Salt Lake City, UT 84112 USA; 2 Department of Biomedical Informatics, School of Medicine, University of Utah, 421 Wakara Way, Salt Lake City, UT 84108 USA; 3 Department of Oncological Sciences, School of Medicine, University of Utah, 2000 Circle of Hope, Salt Lake City, UT 84112 USA; 4 Department of Human Genetics, School of Medicine, University of Utah, 15 South 2030 East, Salt Lake City, UT 84112 USA; 5 Department of Biology, College of Life Sciences, Brigham Young University, Provo, UT 84602 USA; 6 Division of Computational Biomedicine, School of Medicine, Boston University, 72 East Concord Street, Boston, MA 02218 USA; 7 Department of Biomedical Engineering, Oregon Health and Science University, 2730 SW Moody Avenue, Portland, OR 97201 USA; 8 Oregon Center for Spatial Systems Biomedicine, Oregon Health and Science University, 2730 SW Moody Ave, Portland, OR 97201 USA; 9 Department of Genetics and Lineberger Comprehensive Cancer Center, University of North Carolina, 450 West Drive, Chapel Hill, NC 27599 USA; 10 High-Throughput Genomics and Bioinformatic Analysis, Huntsman Cancer Institute, 2000 Circle of Hope, Salt Lake City, UT 84112 USA; 11 Department of Pathology, Huntsman Cancer Hospital, 1950 Circle of Hope Drive, Salt Lake City, UT 84112 USA; 12 Department of Internal Medicine, Pulmonary Division, School of Medicine, University of Utah, 26 North Medical Drive, Salt Lake City, UT 84132 USA; 13 Department of Internal Medicine, Huntsman Cancer Institute, University of Utah, 2000 Circle of Hope, Salt Lake City, UT 84112 USA; 14 Department of Medicine, Oncology Division, Huntsman Cancer Institute, University of Utah, 2000 Circle of Hope Drive, Salt Lake City, UT 84112 USA; 15 Department of Medical Oncology and Therapeutics, City of Hope Comprehensive Cancer Institute, 1218 S Fifth Ave, Monrovia, CA 91016 USA

*Nature Communications* 10.1038/s41467-017-01174-3, Article published online 1 November 2017

The originally published version of this article contained an error in Figure 4. In panel a, grey boxes surrounding the subclones associated with patients #2 and #4 obscured adjacent portions of the heatmap. This has now been corrected in both the PDF and HTML versions of the article.

