# Supplementary material for: Combating subclonal evolution of resistant cancer phenotypes
Source: Nat Commun. 2017 Nov 1;8:1231. doi: 10.1038/s41467-017-01174-3 (PMC5666005; doi:10.1038/s41467-017-01174-3)
Supplement: Supplementary file 1 — Supplementary Information [file 41467_2017_1174_MOESM1_ESM.pdf]

Supplementary Figure 1

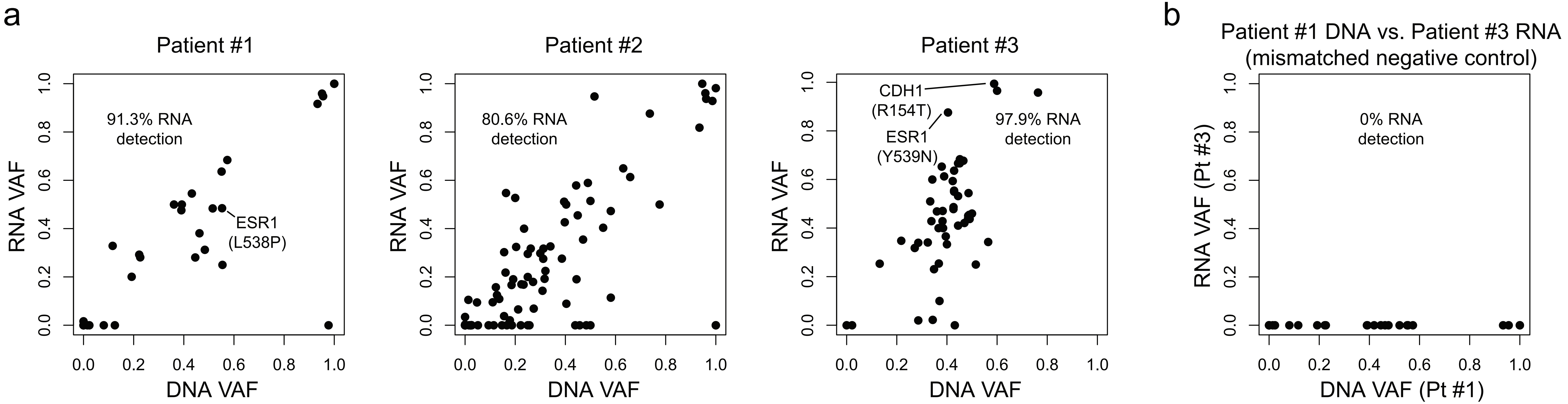

Supplementary Figure 1 | Validation of WGS-detected DNA mutations by RNA-Seq. (**a**) Somatic SNVs detected by WGS (x-axis) were validated in matched bulk RNA-Seq (y-axis) using UNCeqR. VAF indicates variant allele frequency and each point indicates one SNV. Only SNVs in expressed regions (at least 10-read depth in RNA-Seq) were analyzed. Timepoints shown for each patient are day 0 (patient #1), day 0 (patient #2), and day 1163/1168 (patient #3), which were used due to high tumor purity. Bulk RNA-Seq was not performed on patient #4. "RNA detection" percentages refer to the percent of SNVs with DNA VAF above 0.1 that were detected in RNA-Seq. (**b**) As in **a** except that patient #1 DNA mutations were analyzed in patient #3 RNA-Seq as a negative control to determine the specificity of results in **a**.

Supplementary Figure 2

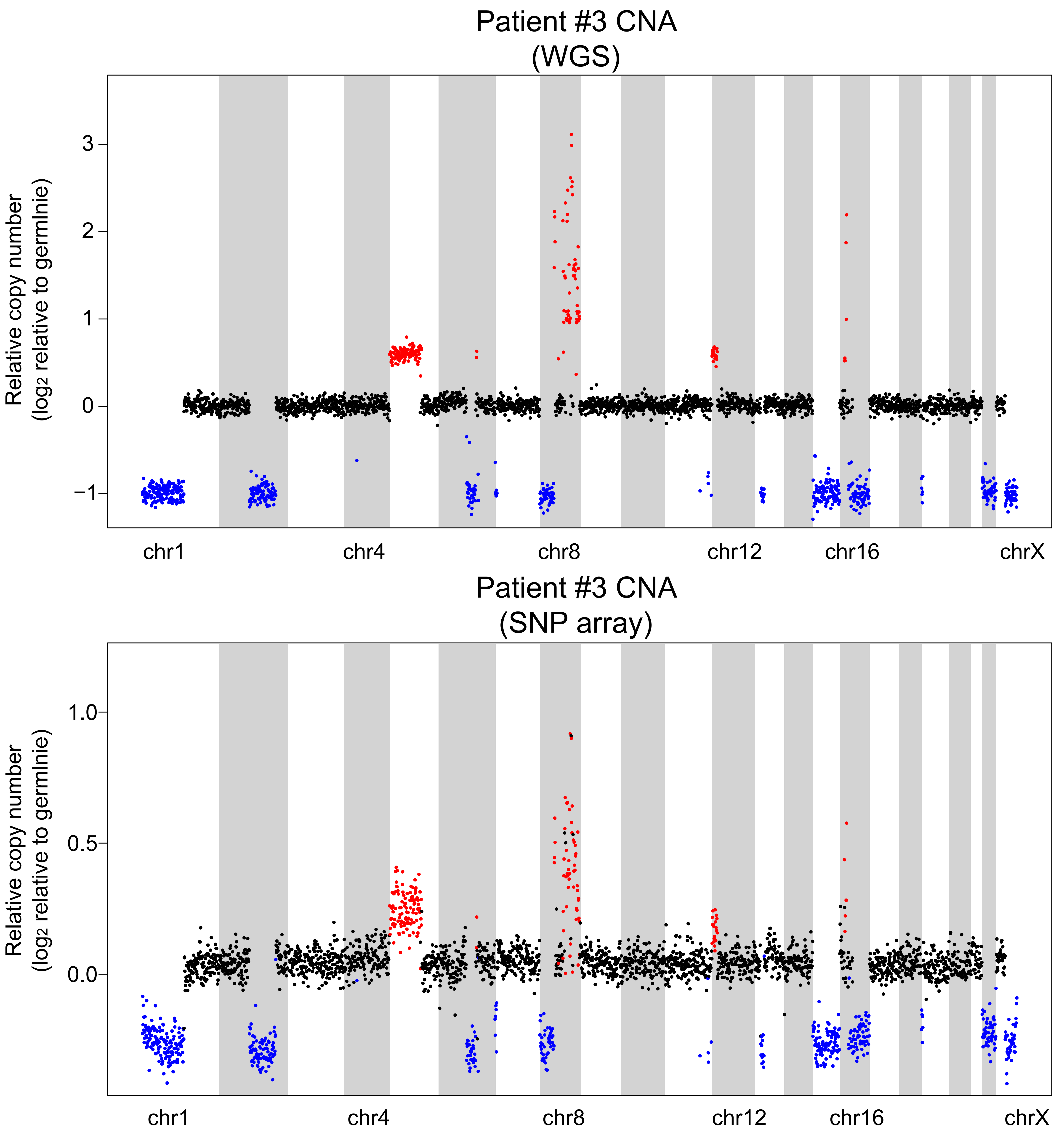

Supplementary Figure 2 | SNP array copy number analysis corroborates WGS copy number analysis. Top, patient #3 day 1168 WGS data were analyzed to identify CNAs relative to the patient's germline. 30-segment window averages are shown (log_2 fold change from germline). Each grey or white region indicates one chromosome. Regions greater than or equal to 0.3 were considered amplified (red) while regions less than or equal to -0.3 were considered deleted (blue). Bottom, copy number analysis was performed on the same sample using a SNP array (Illumina Infinium OmniExpressExome-8 v1.4), and regions highlighted in top panel are highlighted in the same color in the bottom panel, showing corroboration between CNA analysis in WGS vs. SNP array.

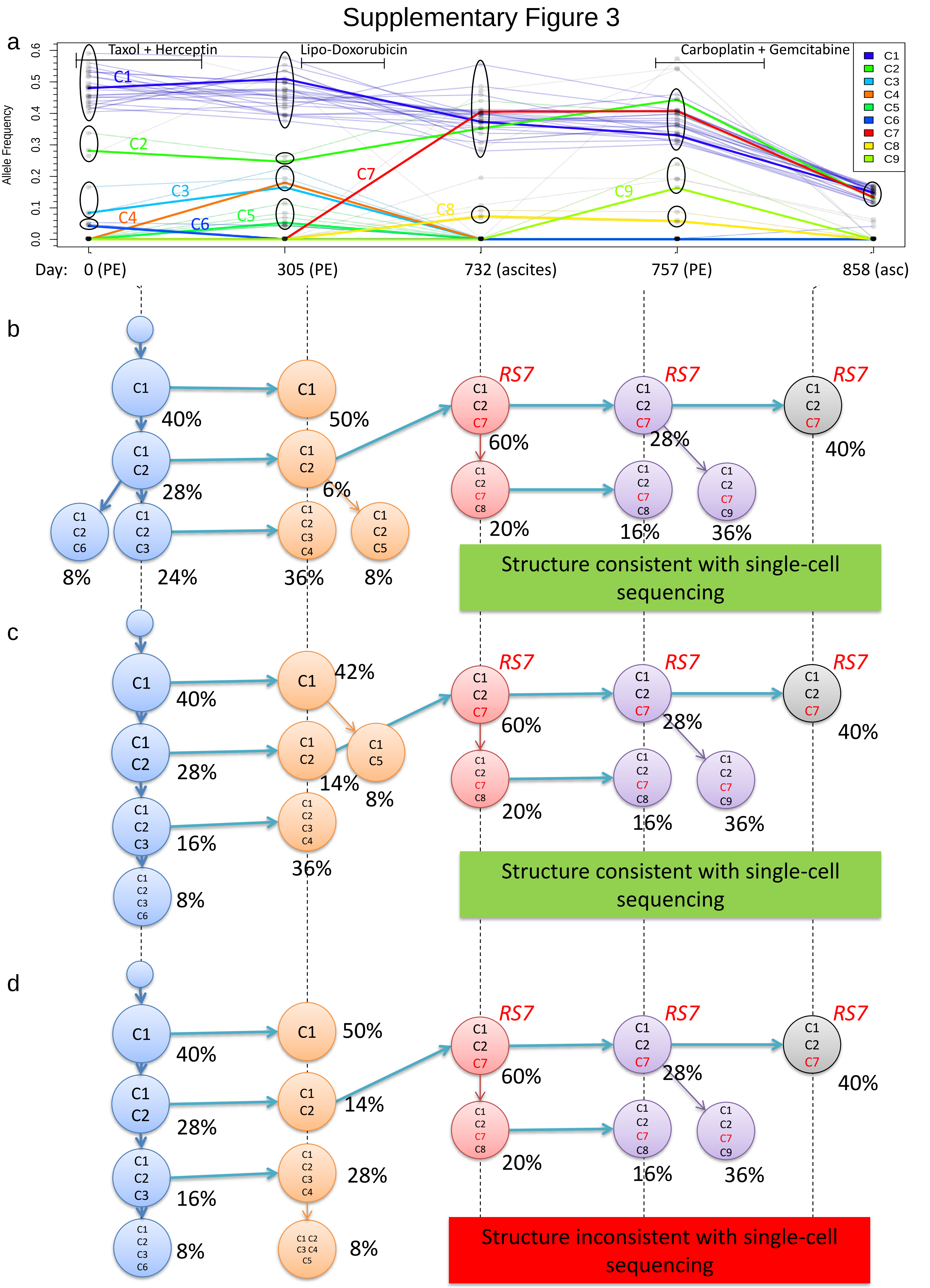

Supplementary Figure 3 | Possible subclonal evolution structures identified by SubcloneSeeker for breast cancer patient #1. (**a**) Variant allele frequencies for coding, diploid mutations identified from exome sequencing. Mutation clusters (C1-C9), identified by Affinity Propagation Clustering, are indicated by colors. (**b-d**) Possible subclonal evolution structures identified through analysis of mutations in (a) with SubcloneSeeker. CCFs are indicated next to each subclone. (b) and (c) were found to be consistent with single-cell sequencing while (d) was not. RS7 indicates resistant (bottleneck) subclone from cluster C7.

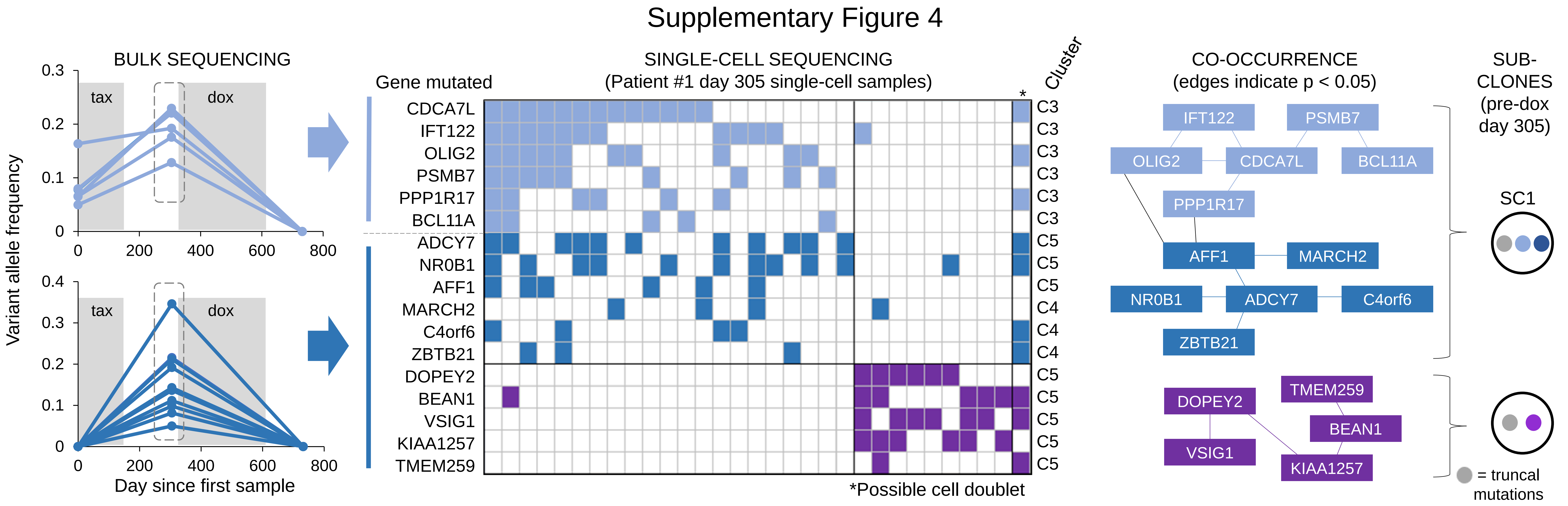

Supplementary Figure 4 | Single-cell sequencing of pre-doxorubicin cells in breast cancer patient #1. Left, bulk variant allele frequencies of mutations increasing after taxane therapy studied by single-cell sequencing as determined by bulk whole-exome sequencing (WES). Middle, results of single-cell sequencing of pre-dox cells (day 305), with each column representing a single cell, mutations sequenced indicated by the name of the gene (or nearby gene) mutated, and mutations indicated by filled squares; mutation clusters identified in Supplementary Figure 3 are also indicated. Right, co-occurrence analysis of mutations in single cells; edges indicated $p < 0.05$ by Fisher's exact test. Subclones from Fig. 2a are indicated for the groups of mutations.

Supplementary Figure 5

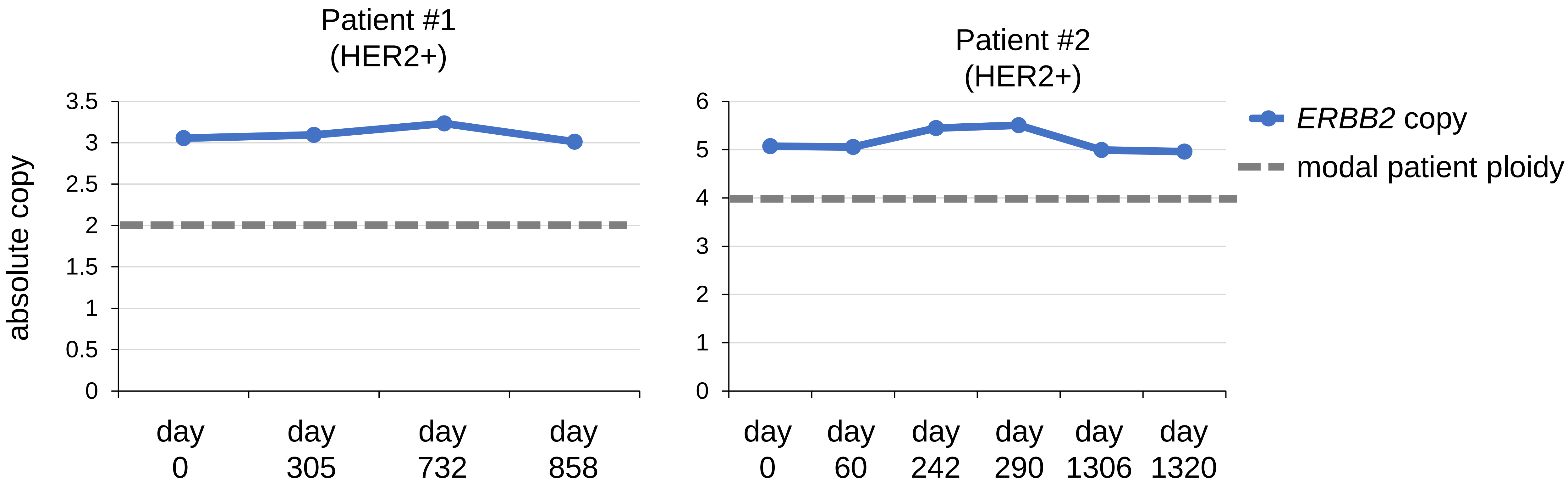

Supplementary Figure 5 | Clinically HER2+ patients also have *ERBB2* genomic amplification. Absolute copy number at the *ERBB2* locus (blue line) based on WGS in patients #1 and #2, both of whom were clinically determined to be HER2+ after cancer diagnosis. Gray dotted line indicates the genome-wide modal copy number in each patient (patient #1 is pseudo-diploid while patient #2 is pseudo-tetraploid) for comparison.

Supplementary Figure 6

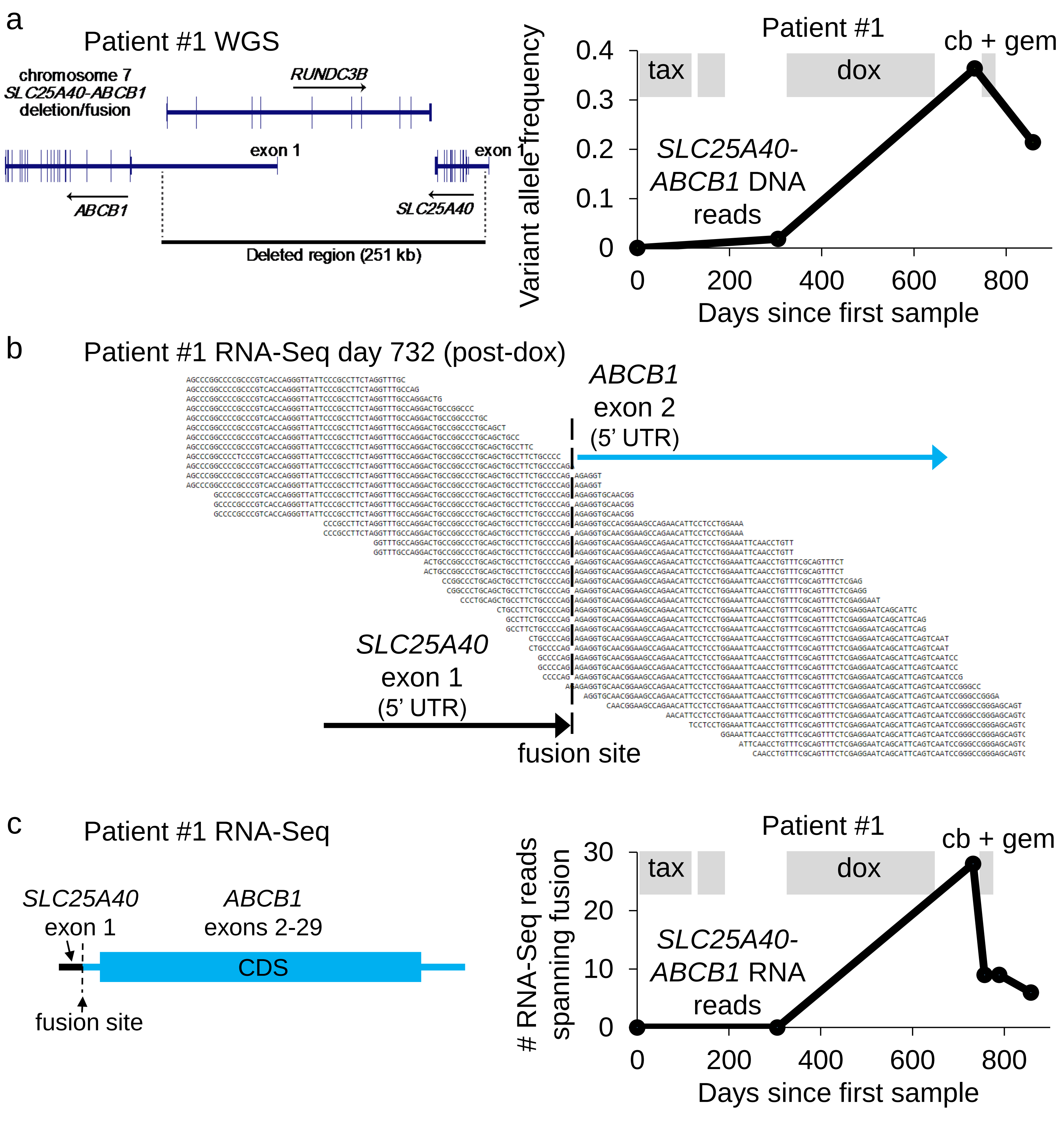

Supplementary Figure 6 | Identification of *SLC25A40-ABCB1* fusion in patient #1 after doxorubicin. (**a**) Structure of *SLC25A40-ABCB1* per WGS (left) and variant allele frequencies over time for the *SLC25A40-ABCB1* fusion (right) in patient #1. (**b**) Schematic of split RNA-Seq reads from post-doxorubicin (day 732) sample. (**c**) Inferred *SLC25A40-ABCB1* fusion transcript (left) and number of RNA-Seq reads (reads spanning plus mate pairs spanning) supporting the *SLC25A40-ABCB1* fusion at each timepoint (right).

Supplementary Figure 7

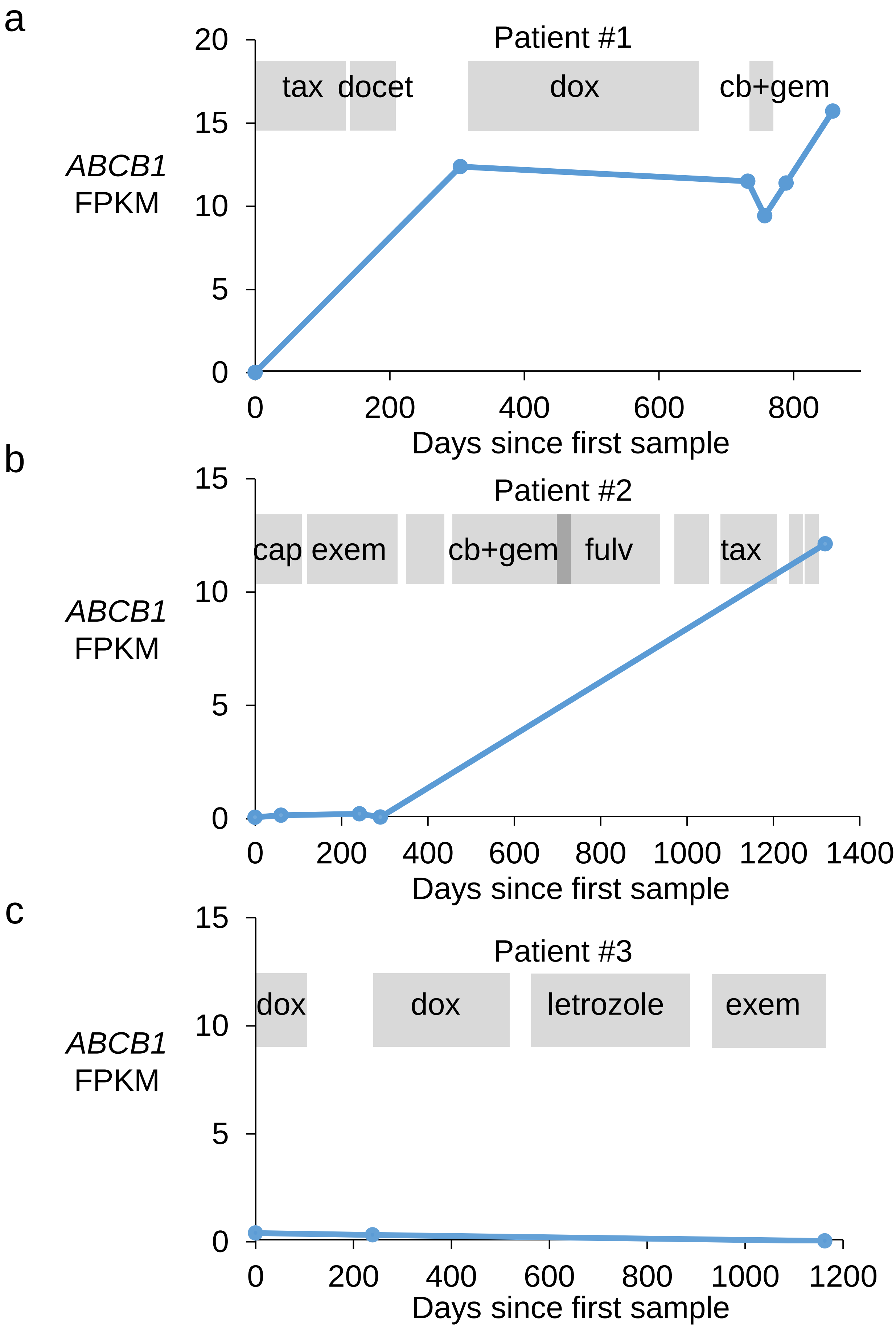

Supplementary Figure 7 | Evolution of *ABCB1* expression in three breast cancer patients. *ABCB1* FPKM (RNA-Seq) for patients #1 (**a**), #2 (**b**), and #3 (**c**) over time with drug treatment indicated in grey. See Fig. 2 legend for drug abbreviations.

Supplementary Figure 8

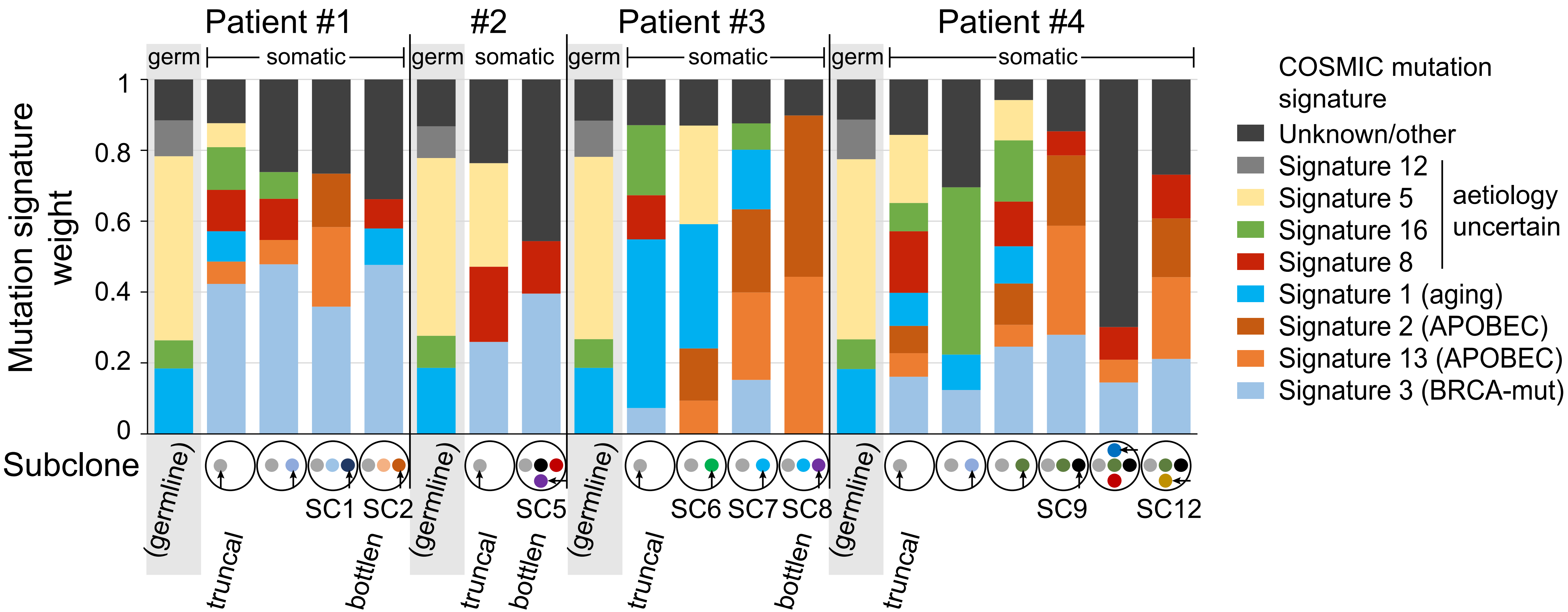

Supplementary Figure 8 | APOBEC and BRCA-deficiency mutation signatures are found in somatic but not germline variants. Mutation signature weights for COSMIC mutation signatures in indicated somatic SNV clusters or germline ("germ") SNPs (as a control) identified by WGS. Signatures with less than 0.03 average weight (except for signature 12) and mutations not accounted for by known signatures are included in the unknown/other group.

Supplementary Figure 9

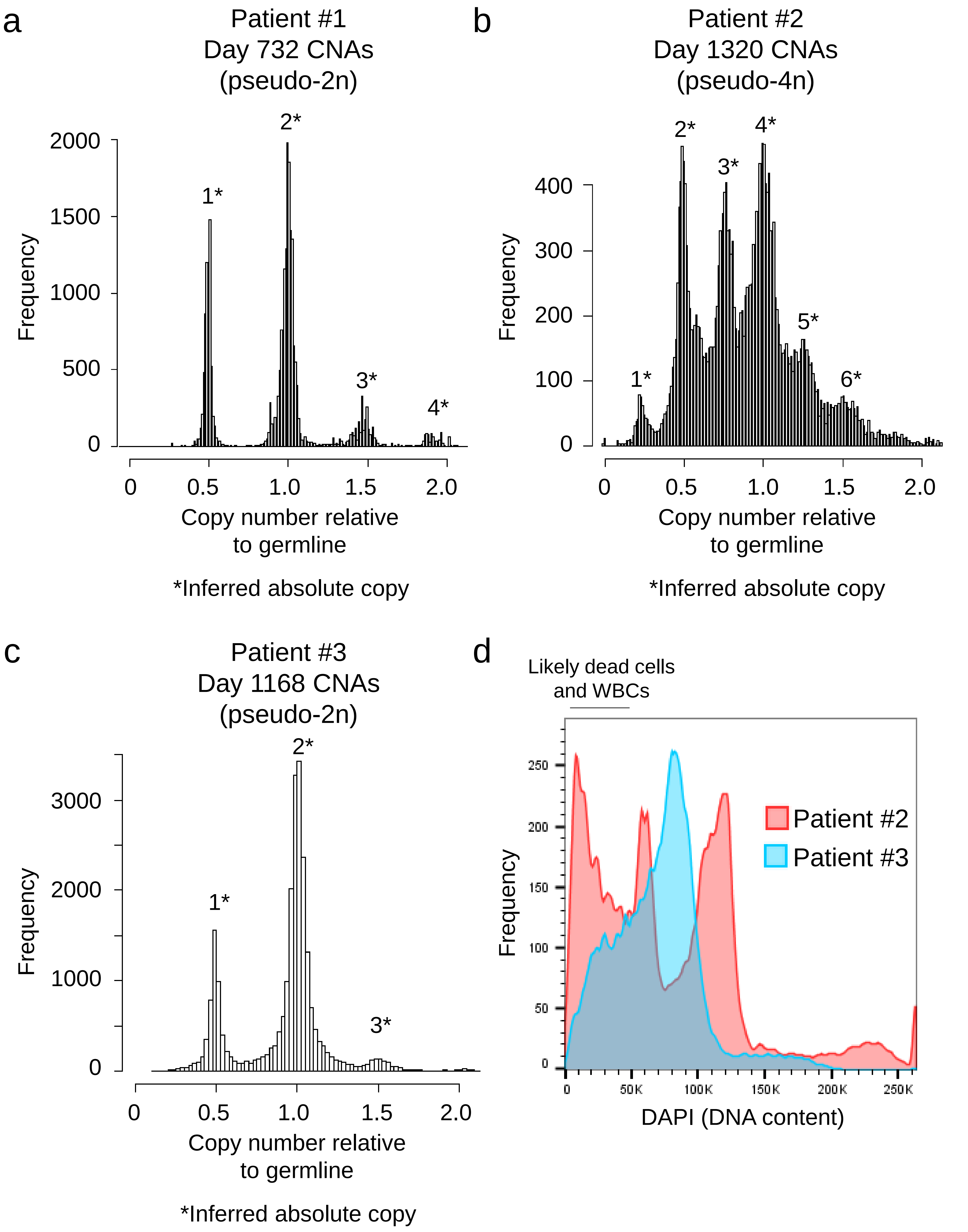

Supplementary Figure 9 | Patient #2 breast cancer is pseudo-tetraploid. Copy number values relative to germline for each gene, inferred from WGS, are shown in histogram format for indicated samples from patients #1 (**a**), #2 (**b**), and #3 (**c**). Inferred absolute integer copy is indicated with asterisks by peaks. (**d**) Flow cytometry was performed on example samples from patients #2 (red) and #3 (light blue) using DAPI to quantify DNA content.

Supplementary Figure 10

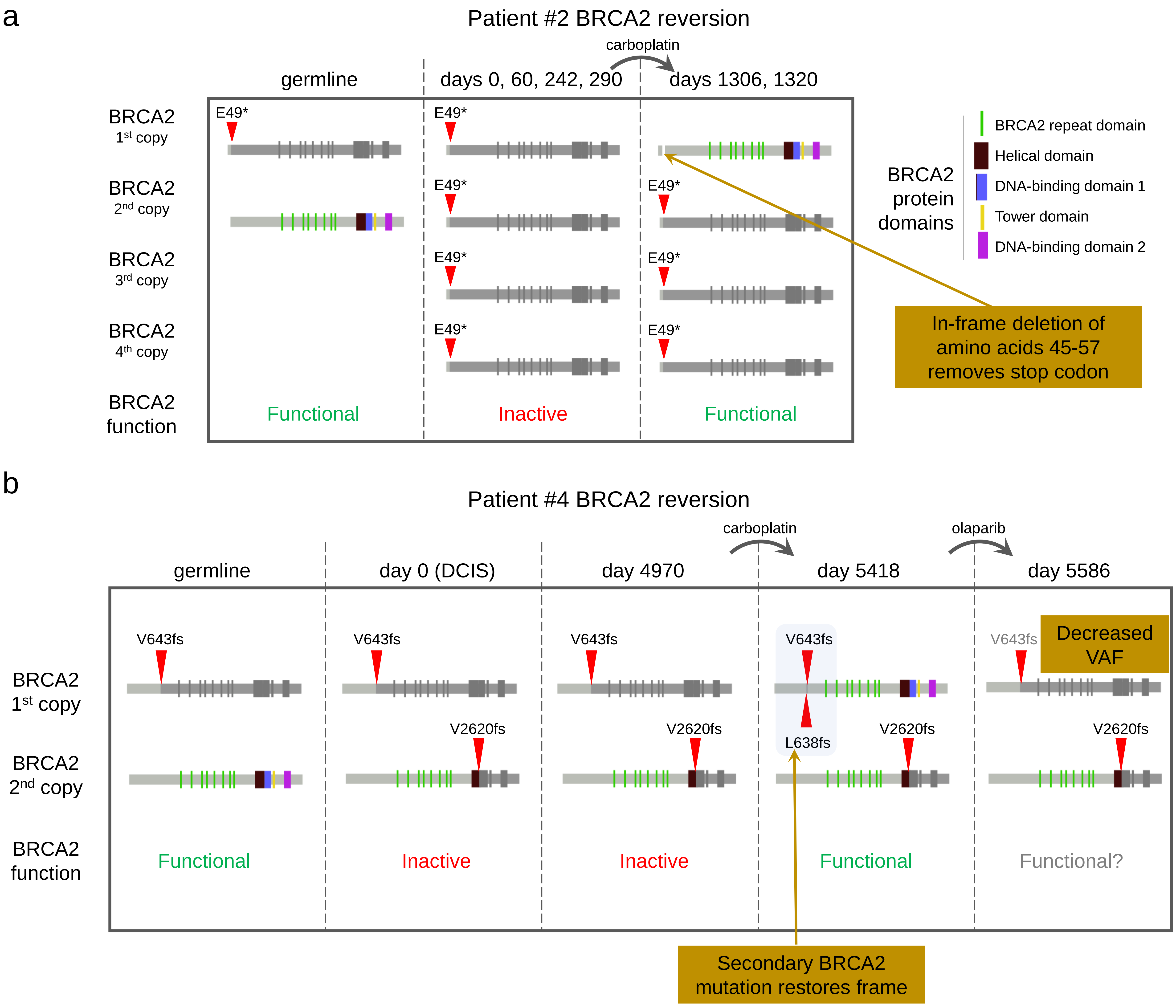

Supplementary Figure 10 | *BRCA2* reversions after platinum treatment in two breast cancer patients. Schematics showing mutation status of *BRCA2* in patients #2 (**a**) and #4 (**b**). BRCA2 protein domain structure is from N- (left) to C-terminus (right), with mutations indicated as red arrows. Copy number for *BRCA2* was inferred as described in Methods, and number of copies of each mutation was inferred using mutation allele frequencies. “Decreased VAF” indicates decreased variant allele frequency of the V643fs germline variant in patient #4, representing a possible direct reversion in some subclone(s).

Supplementary Figure 11

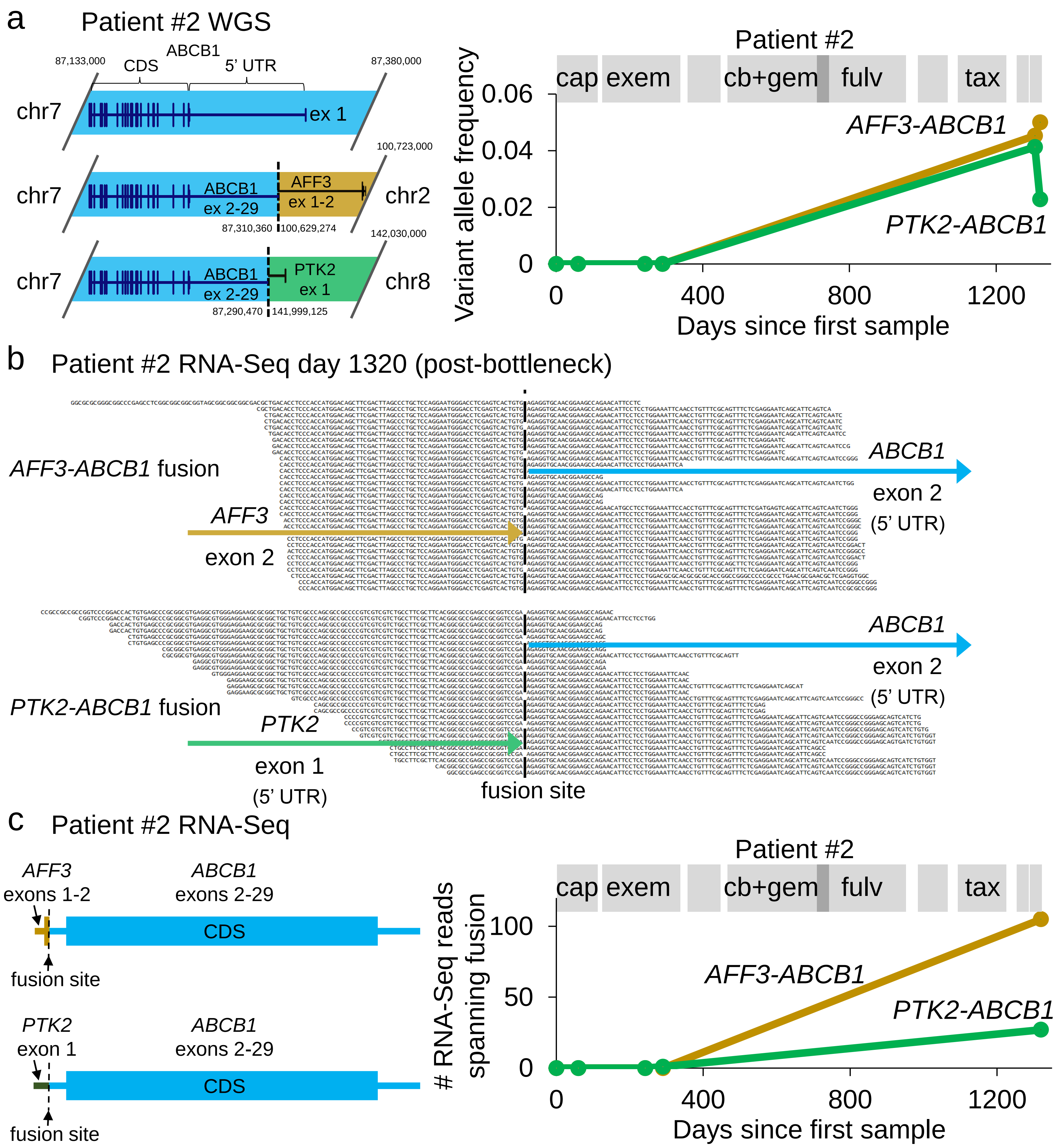

Supplementary Figure 11 | Identification of *AFF3-ABCB1* and *PTK2-ABCB1* fusions in patient #2 after treatment. (**a**) Structure of *AFF3-ABCB1* and *PTK2-ABCB1* fusions per WGS (left) and their variant allele frequencies over time (right) in patient #2. (**b**) Schematic of split RNA-Seq reads from post-bottleneck (day 1320) sample. Reverse complements of anti-sense reads are shown for clarity. (**c**) Inferred *AFF3-ABCB1* and *PTK2-ABCB1* fusion transcripts (left) and number of RNA-Seq reads (split reads) supporting these fusions at each timepoint (right).

Supplementary Figure 12

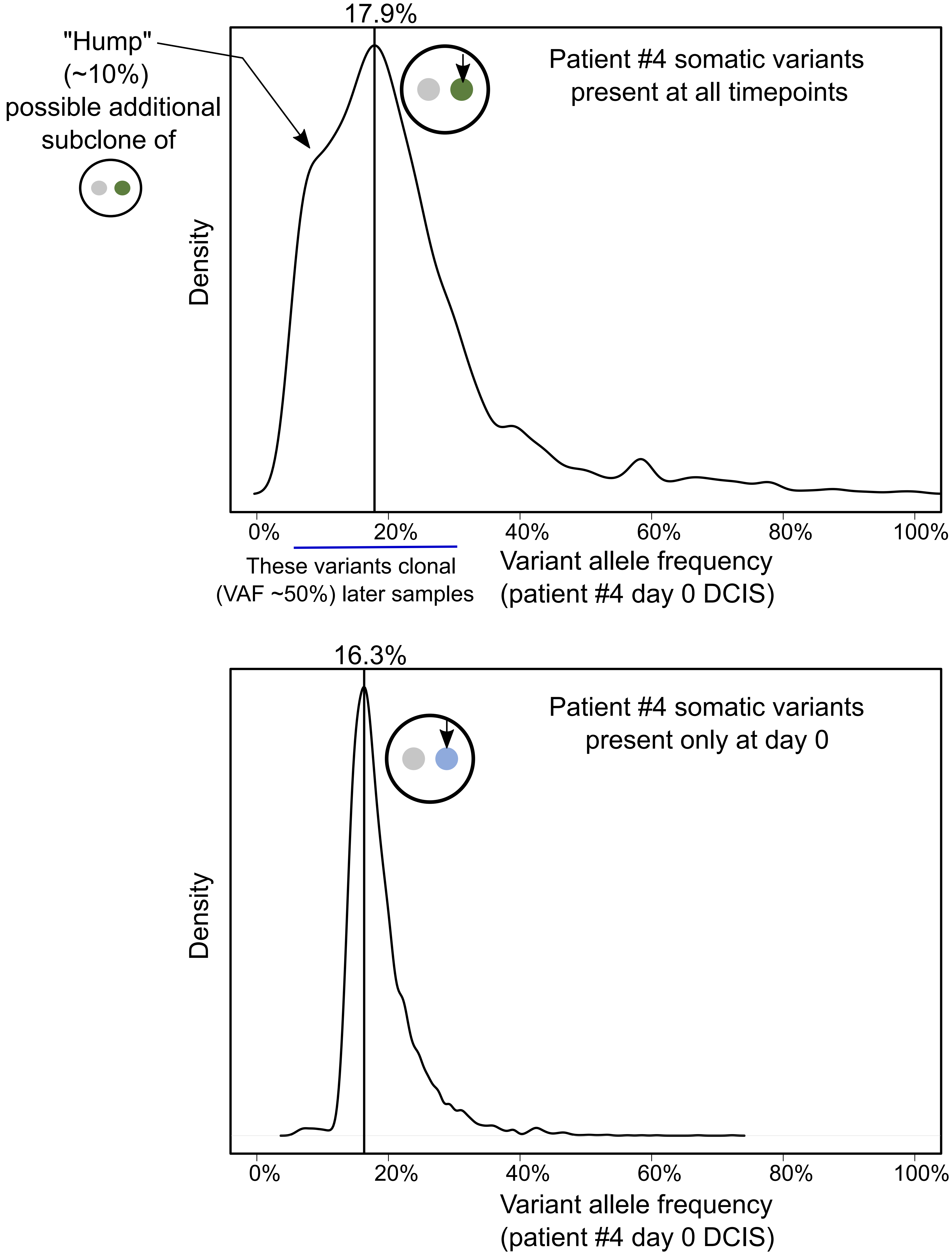

Supplementary Figure 12 | Subclonal analysis of patient #4 day 0 sample. Kernel density plots of copy-neutral somatic mutations (SNVs, indels) in the patient #4 day 0 (DCIS) sample. x-axis represents variant allele frequencies for these mutations. Top shows mutations present at all cancer timepoints with VAF above 0.05, while bottom shows mutations present only at day 0. The mutation cluster and subclone are indicated for each peak. VAFs at peak maxima are indicated above peaks. "Hump" peak indicates possible (surviving) subclone of cells within subclone containing dark green mutations.

Supplementary Figure 13

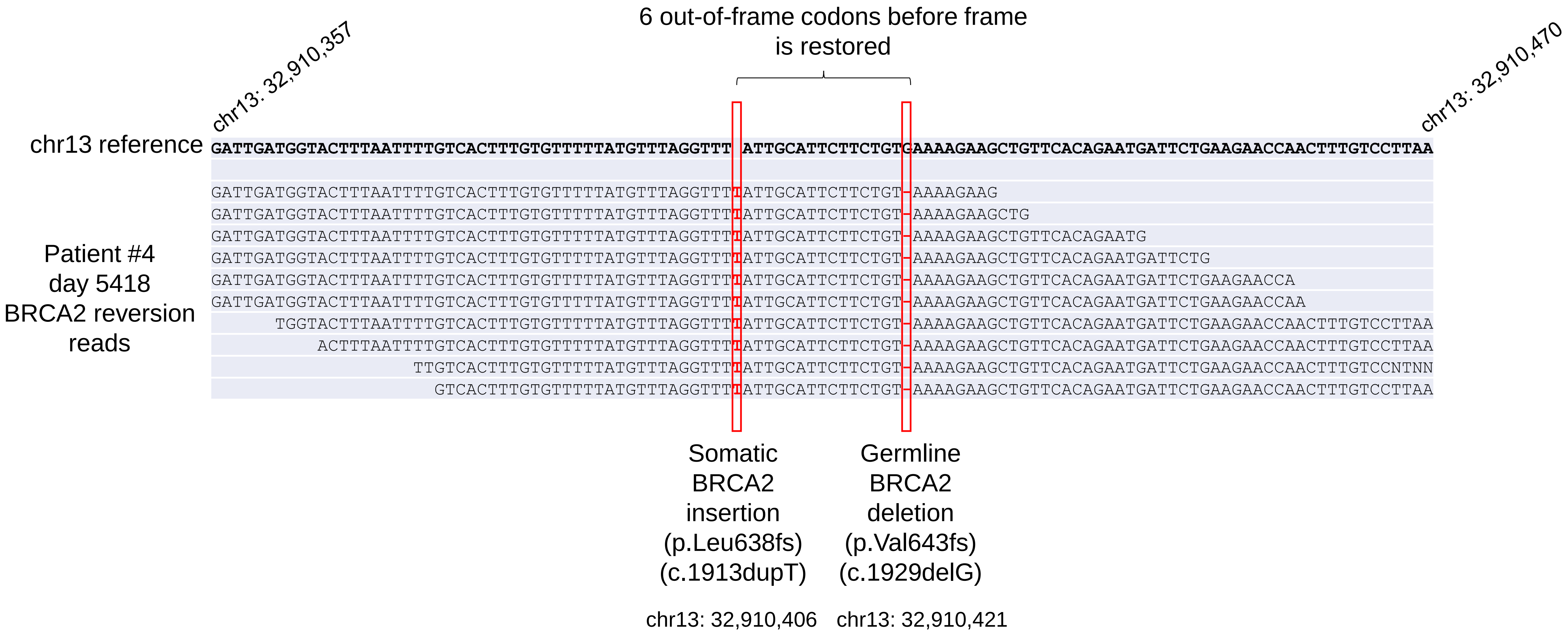

Supplementary Figure 13 | *BRCA2* germline mutation and reversion are in *cis*, not *trans*, in patient #4. WGS reads showing germline *BRCA2* deletion and somatic *BRCA2* insertion are on the same copy of *BRCA2* (on the same reads), thus indicating a restoration of frame (*BRCA2* reversion), in patient #4 day 5418 cancer sample. For some reads the reverse complement of the read sequence is shown. Human reference genome chromosome 13 region is shown above patient sample reads for comparison. Dash indicates deleted nucleotide.

Supplementary Figure 14

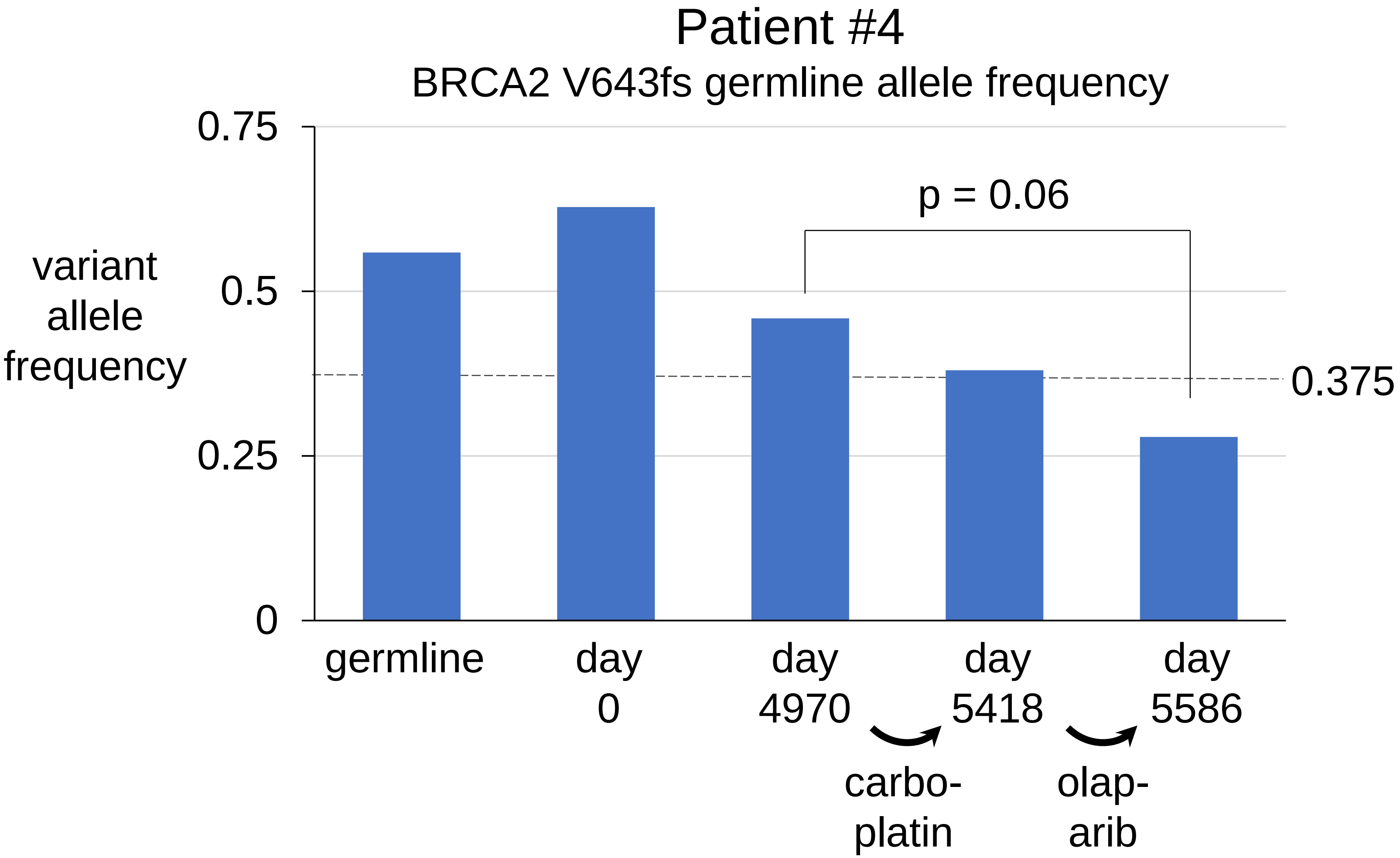

Supplementary Figure 14 | Possible direct *BRCA2* reversion in patient #4 at last timepoint. Variant allele frequencies of patient #4 *BRCA2* germline mutation (V643fs) at each timepoint. P-value is by two-sample proportion test for number of mutant vs. total reads.

Supplementary Figure 15

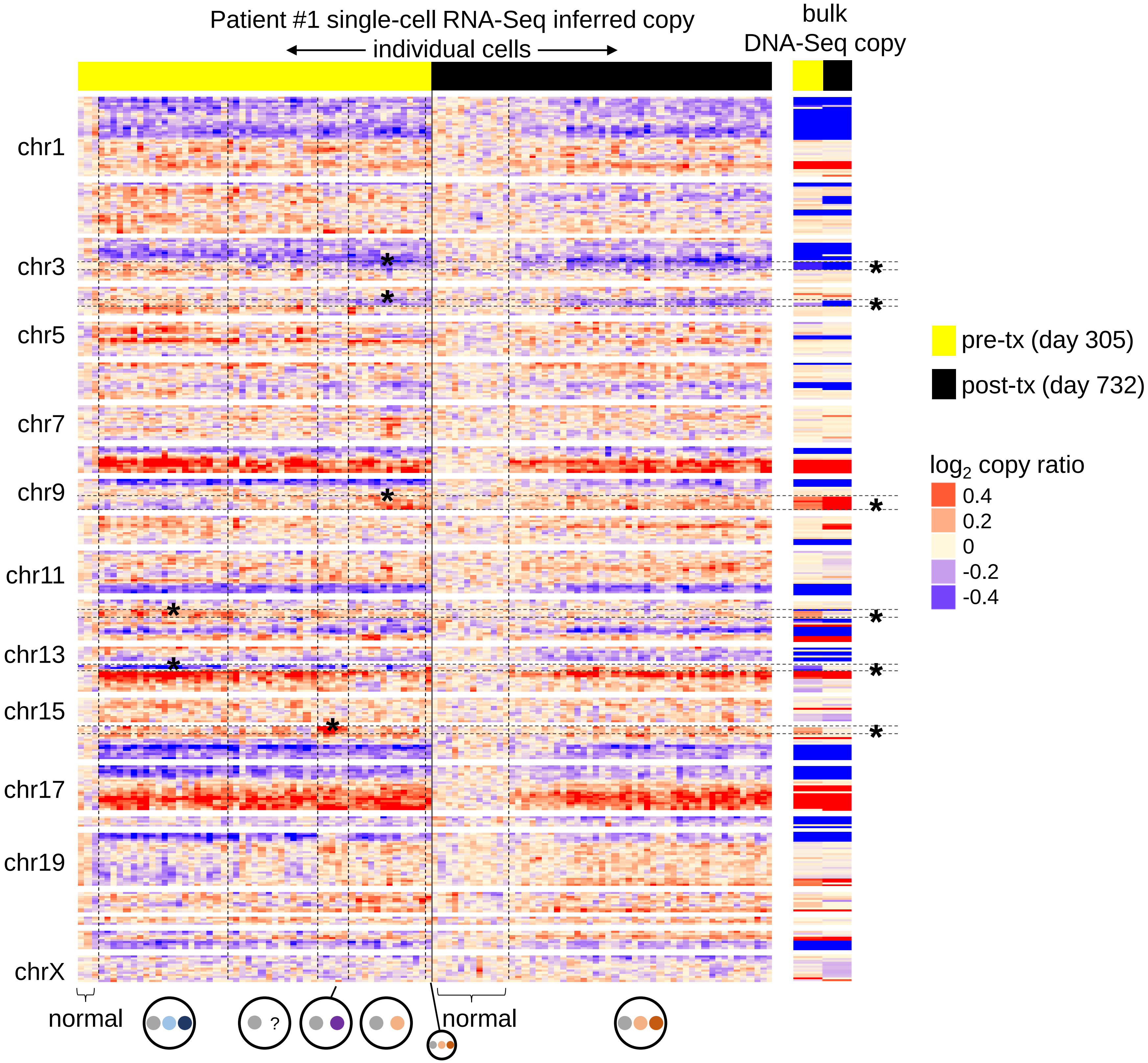

Supplementary Figure 15 | Copy number and subclones inferred from single-cell RNA-Seq of patient #1. scRNA-Seq was performed on pre- and post-treatment cells from patient #1 and copy number was inferred and corroborated with bulk DNA-Seq-based copy for each gene. Subclones were identified using CNAs as described in text and Methods. Asterisks (*) are shown above CNAs aiding in subclone identification (chromosomes 3, 4, 9, 12, 14,and 16) and to the right of the heatmap. ? indicates cells with unclear subclone identity. Subclones assigned (bottom) are defined as in Fig. 2a.

Supplementary Figure 16

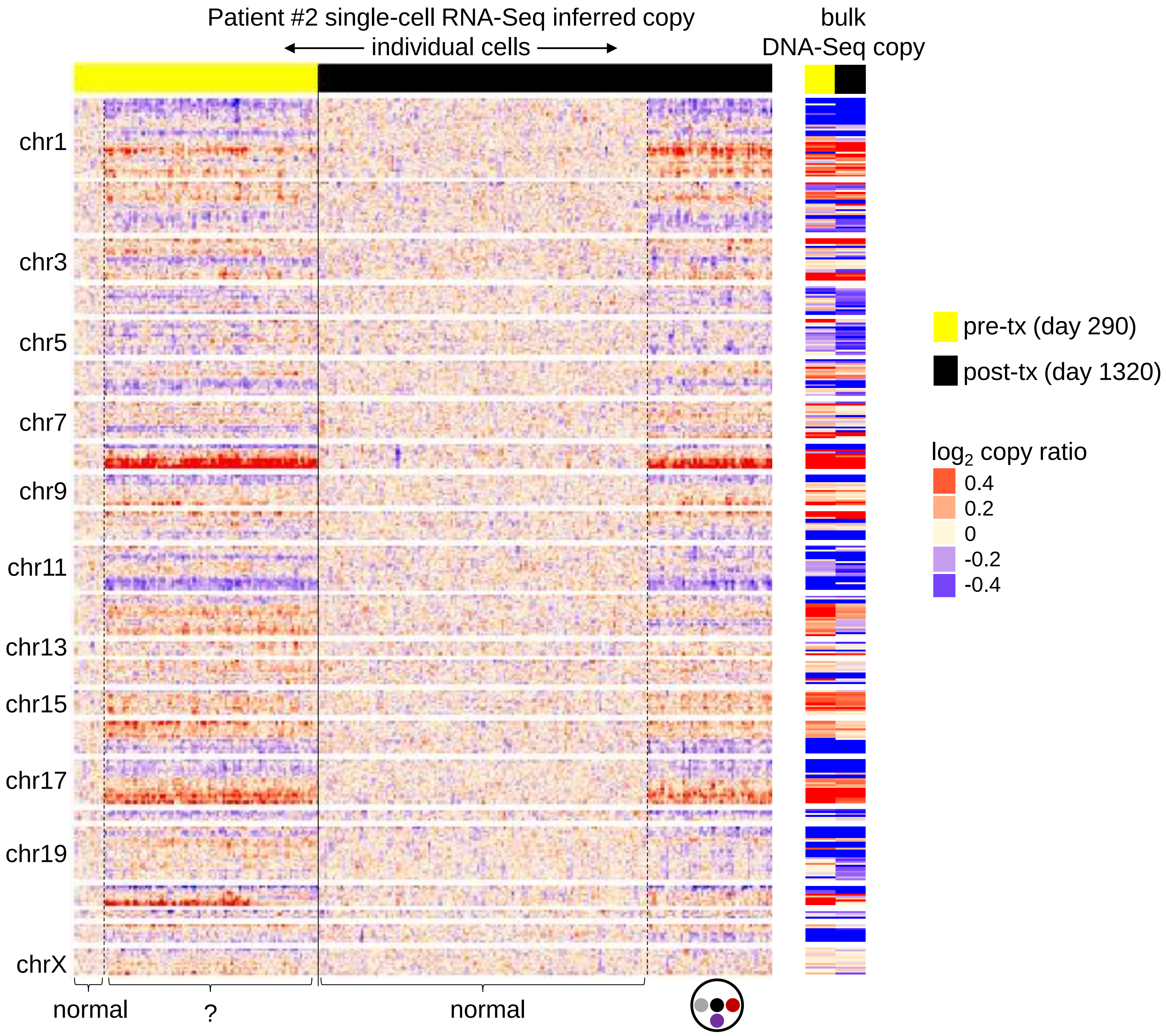

Supplementary Figure 16 | Copy number inferred from single-cell RNA-Seq of patient #2. scRNA-Seq was performed on pre- and post-treatment cells from patient #2 and copy number was inferred and corroborated with bulk DNA-Seq-based copy for each gene, enabling identification of cancer vs. normal cells. “?” indicates cancer cells from unclear subclone.

Supplementary Figure 17

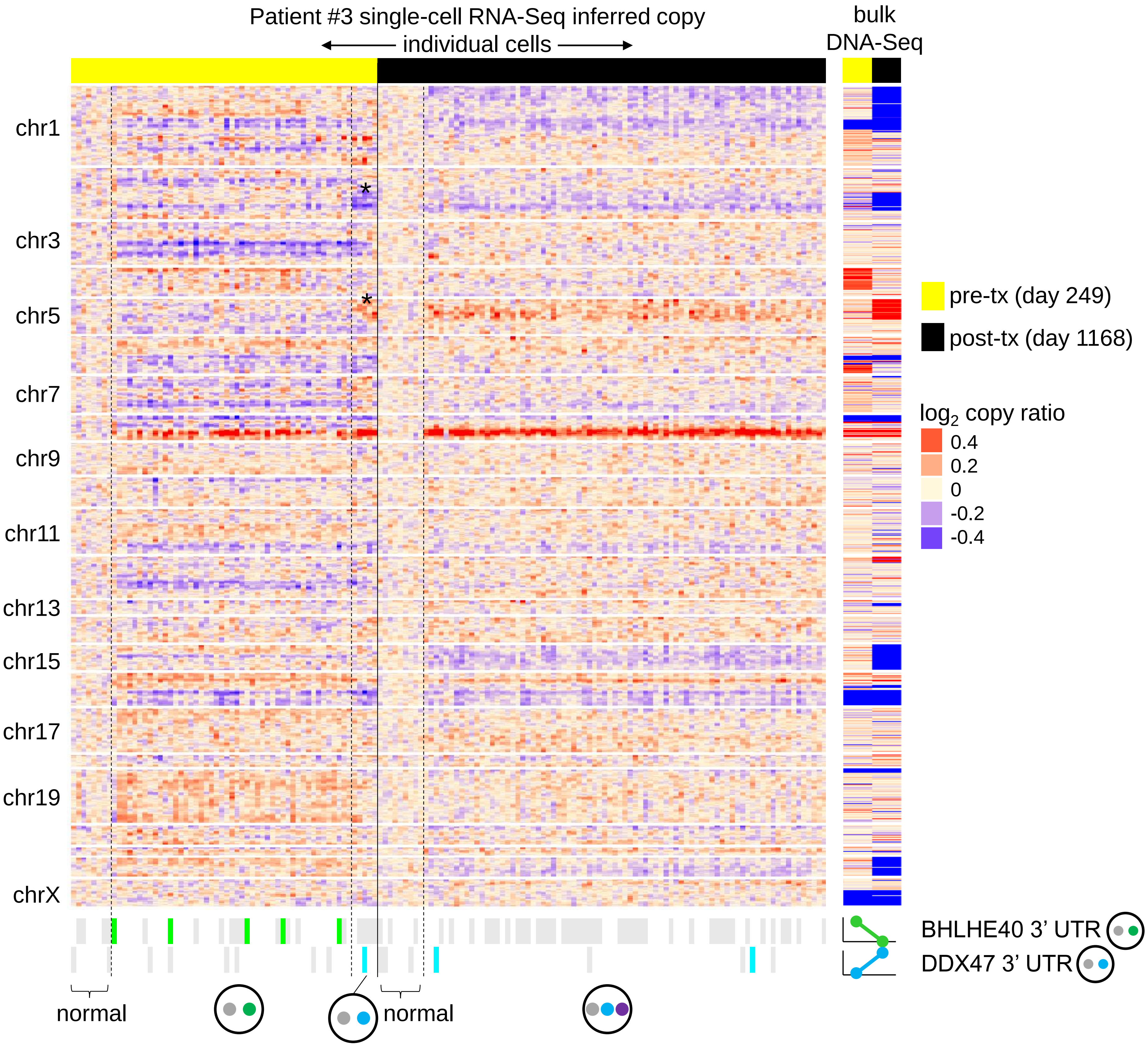

Supplementary Figure 17 | Copy number and subclones inferred from single-cell RNA-Seq of patient #3. scRNA-Seq was performed on pre- and post-treatment cells from patient #3 and copy number was inferred and corroborated with bulk DNA-Seq-based copy for each gene. Subclones were identified using CNAs as described in text and Methods. * indicates CNAs aiding with subclone identification (chromosome 2q deletion and 5p amplification). Subclones assigned (bottom) are defined as in Fig. 2c. At bottom, mutation status of two subclone-specific SNVs (*BHLHE40* and *DDX47* genes) with highest read coverage in scRNA-Seq data are shown as confirmation of subclone assignments inferred by copy number; colored (green and turquoise) hashes indicate mutation present (VAF above 0.1) while grey indicates not present. White indicates lack of coverage. Bulk VAFs of these mutations are indicated in the "bulk DNA-Seq" column (bottom) with the y-axis representing VAF (axis 0 to 0.5). Pre-treatment VAF of DDX47 was 0.03, indicating it would be expected in a minor subclone pre-treatment, and 0.45 after treatment.

Supplementary Figure 18

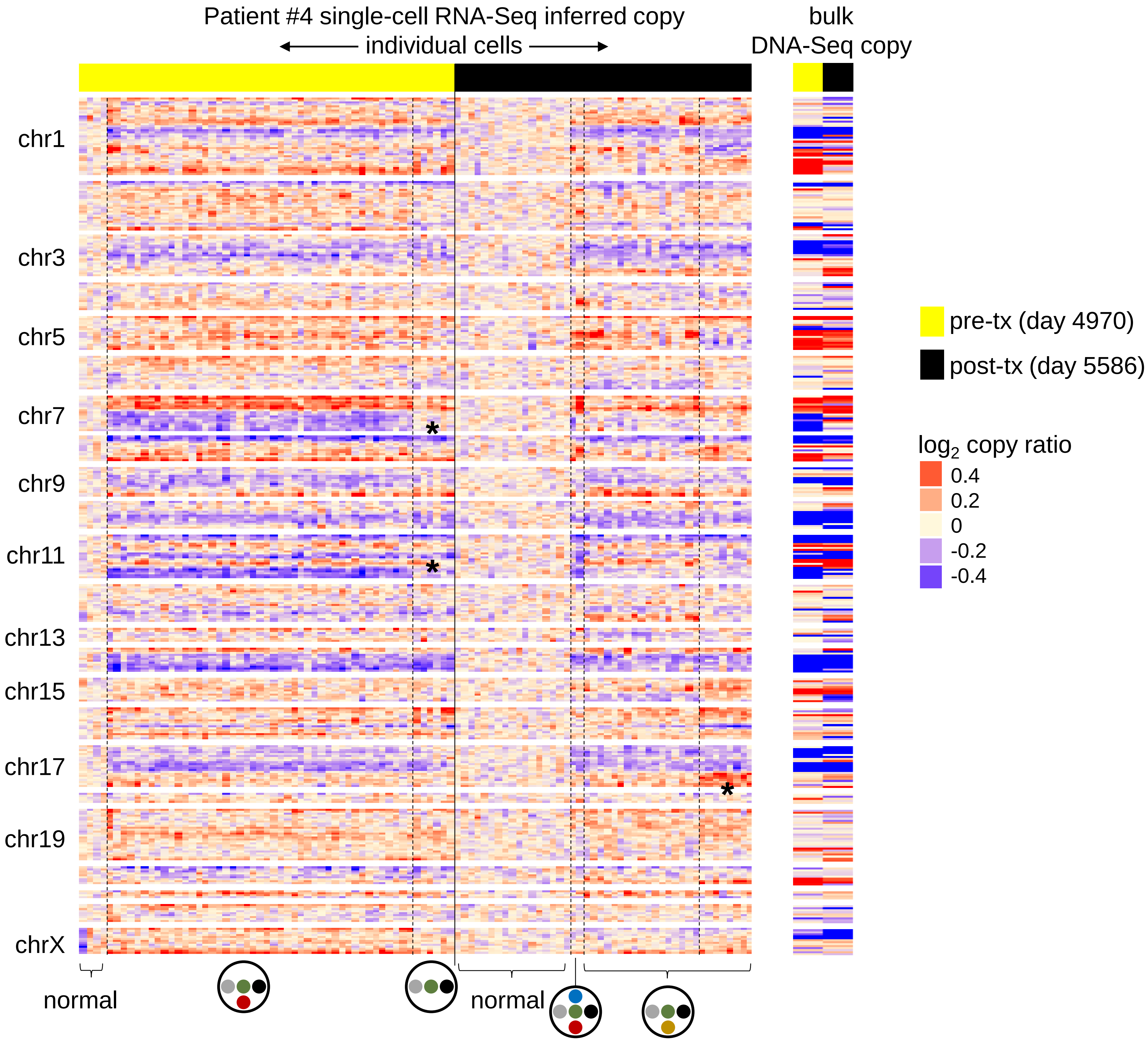

Supplementary Figure 18 | Copy number and subclones inferred from single-cell RNA-Seq of patient #4. scRNA-Seq was performed on pre- and post-treatment cells from patient #4 and copy number was inferred and corroborated with bulk DNA-Seq-based copy for each gene. Subclones were identified using CNAs as described in text and Methods. * indicates CNAs aiding with subclone identification. Subclones assigned (bottom) are defined as in Fig. 2d.

Supplementary Figure 19

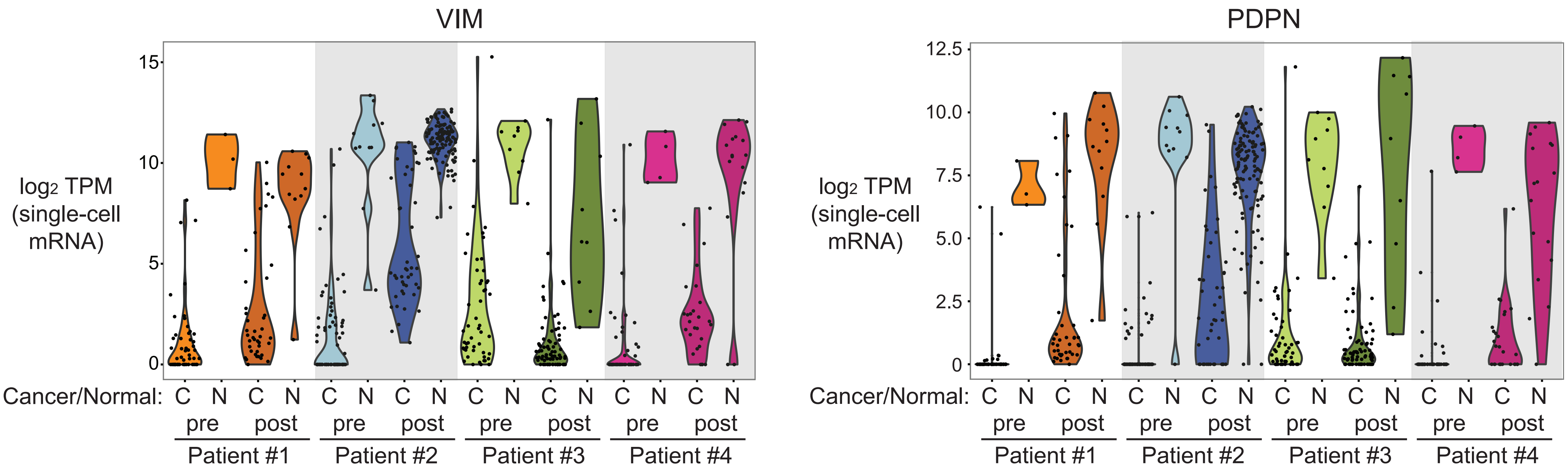

Supplementary Figure 19 | Increased expression of mesenchymal and mesothelial markers in normal cells by single-cell RNA-Seq. Expression of the mesenchymal/fibroblast marker vimentin (*VIM*) and the mesothelial marker podoplanin (*PDPN*) in normal ("N") vs. cancer ("C") cells per scRNA-Seq (violin plot). Normal cells were identified based on lack of copy number alterations inferred from scRNA-Seq. Y-axis represents $\log_2$(TPM + 1) and each point represents one cell. "Pre" and "post" refer to pre- and post-treatment samples.

Supplementary Figure 20

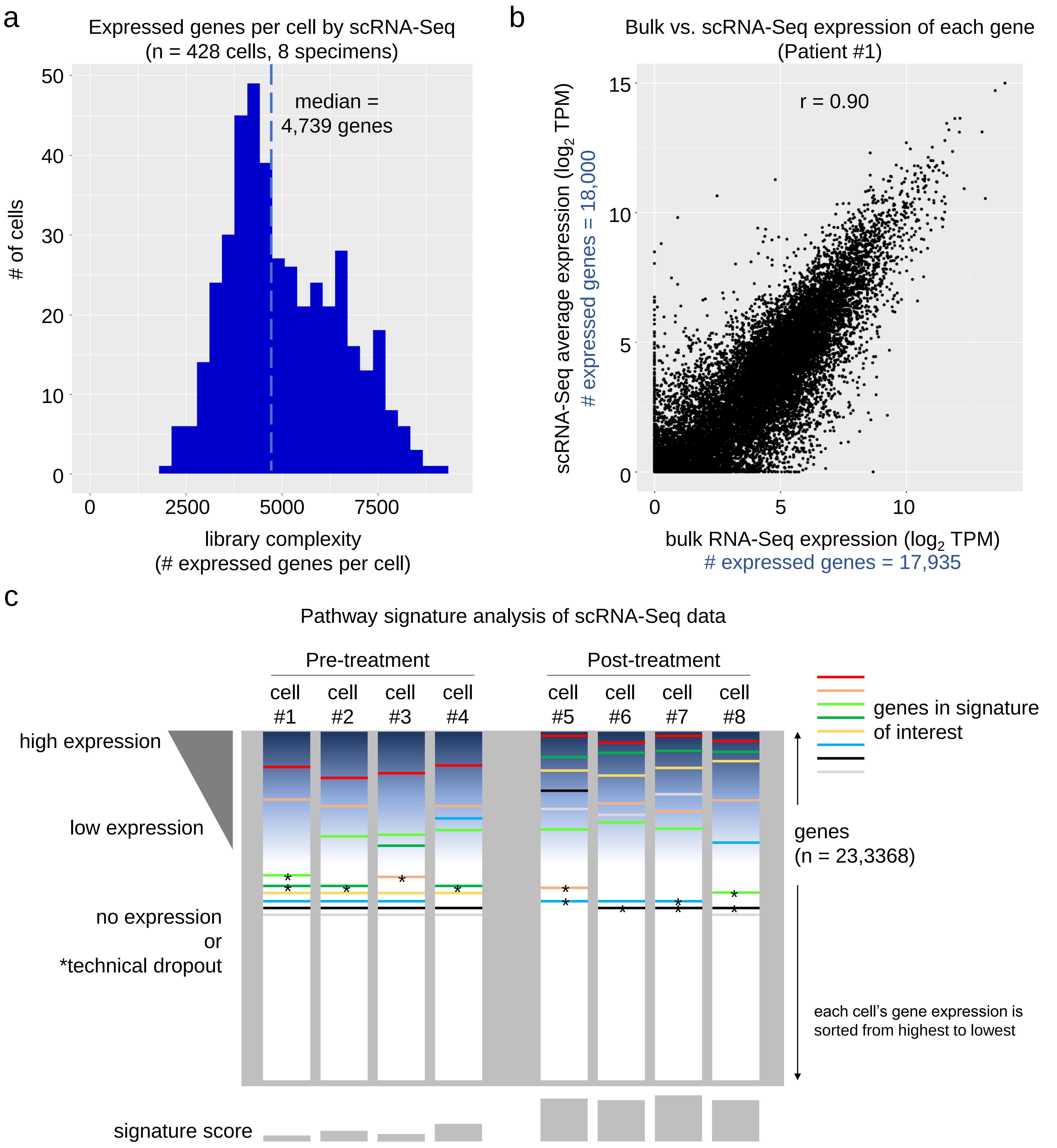

Supplementary Figure 20 | scRNA-Seq quality metrics and pathway analysis approach. (**a**) Histogram showing the number of genes expressed in each of 428 individual cancer cells from 8 breast cancer samples (from 4 patients) by scRNA-Seq. (**b**) Positive correlation between bulk RNA-Seq and scRNA-Seq expression data. The patient #1 day 305 sample was subjected to bulk RNA-Seq (x-axis) and scRNA-Seq (y-axis). For y-axis, average TPM value of each gene was determined for 52 individual cancer cells and values were then log-transformed ($\log_2$(TPM) + 1). Each point represents one gene. r value is by Pearson correlation. Expressed gene number is the number of genes with non-zero gene expression. (**c**) Schematic showing how pathway signature analysis using ssGSEA or ASSIGN mitigates problems of technical gene dropout in scRNA-Seq data, enabling biological interpretation and comparison between pre- and post-treatment samples. Measurement of multiple genes within a pathway produces a composite pathway score that is more stable in the face of technical noise than individual genes. Horizontal colored lines indicate genes belonging to a pathway signature, though most signatures contain many more genes than indicated.

Supplementary Figure 21

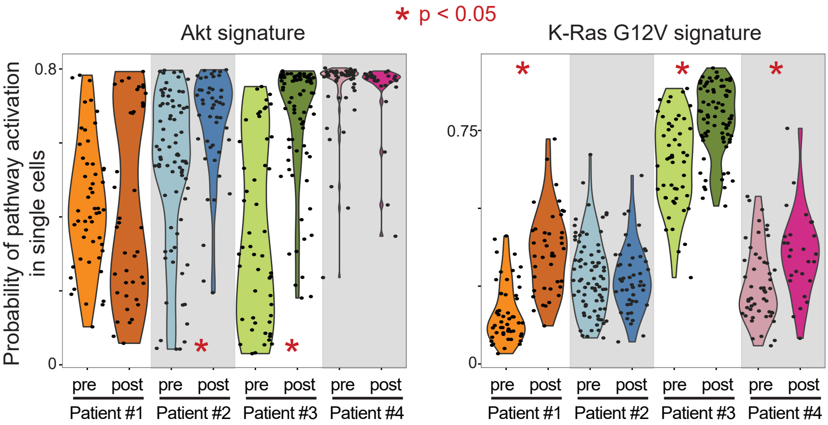

Supplementary Figure 21 | Increased Akt and K-Ras pathway signatures in post-treatment breast cancers. ASSIGN signature scores for Akt and K-Ras G12V signatures (see Methods for signature generation) in individual cancer cells from four breast cancers, before and after treatment. Data are based on scRNA-Seq. * indicates $p < 0.05$ by student's t-test.

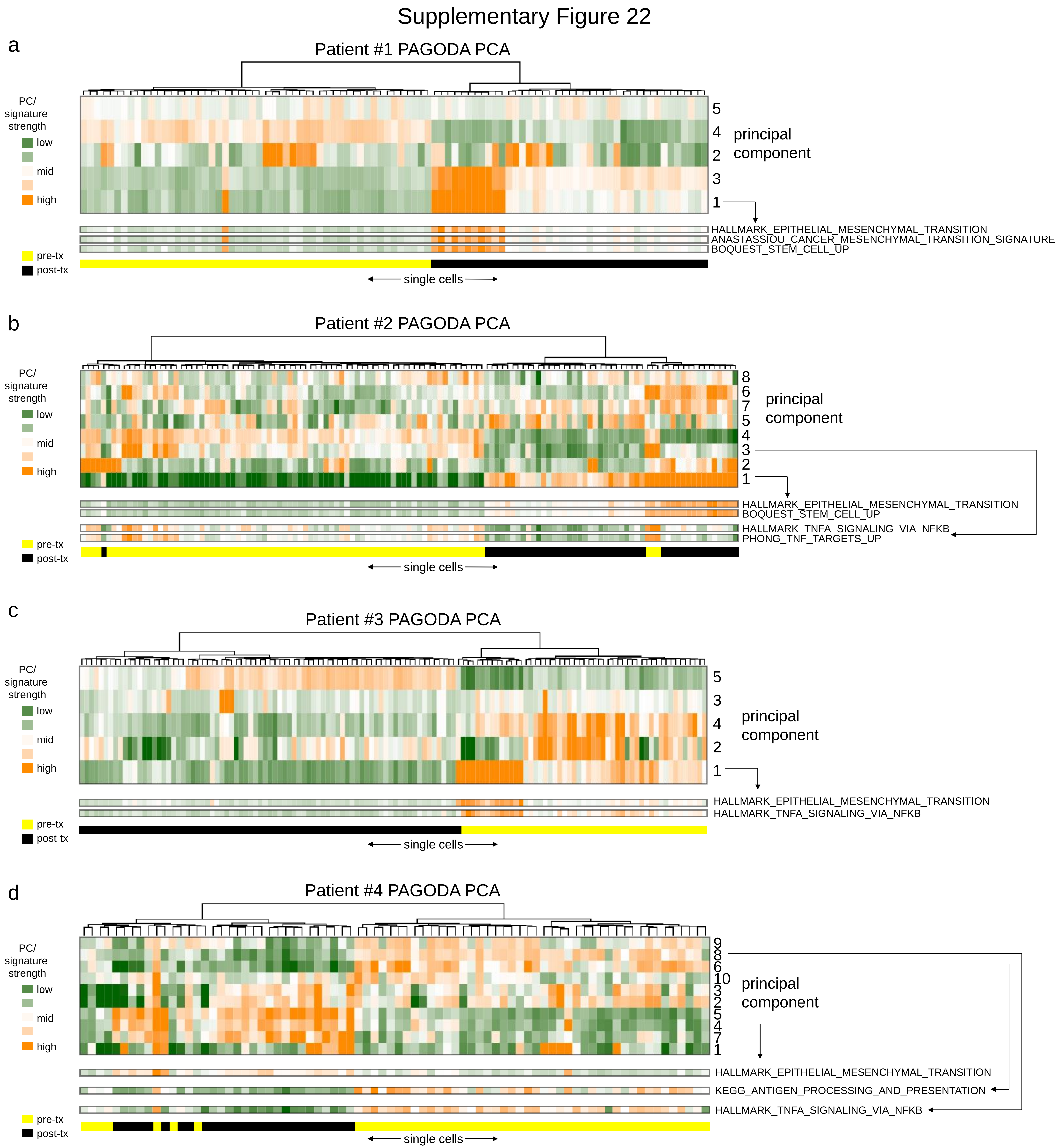

Supplementary Figure 22 | Confirmation of pathways changing between pre- and post-treatment using PAGODA-based signature over-dispersion analysis. PAGODA-based principal component analysis was performed on scRNA-Seq data for patients #1-#4 (**a-d**), using Molecular Signatures Database signatures as input, to identify signatures most variable between cells within each patient. Selected signatures identified in Fig. 5b and c as being statistically different between pre- and post-treatment samples using our primary analysis method (ssGSEA) are indicated below each principal component plot with their principal component indicated by arrows. Principal component and signature strengths are indicated by color (orange, high; green, low).

Supplementary Figure 23

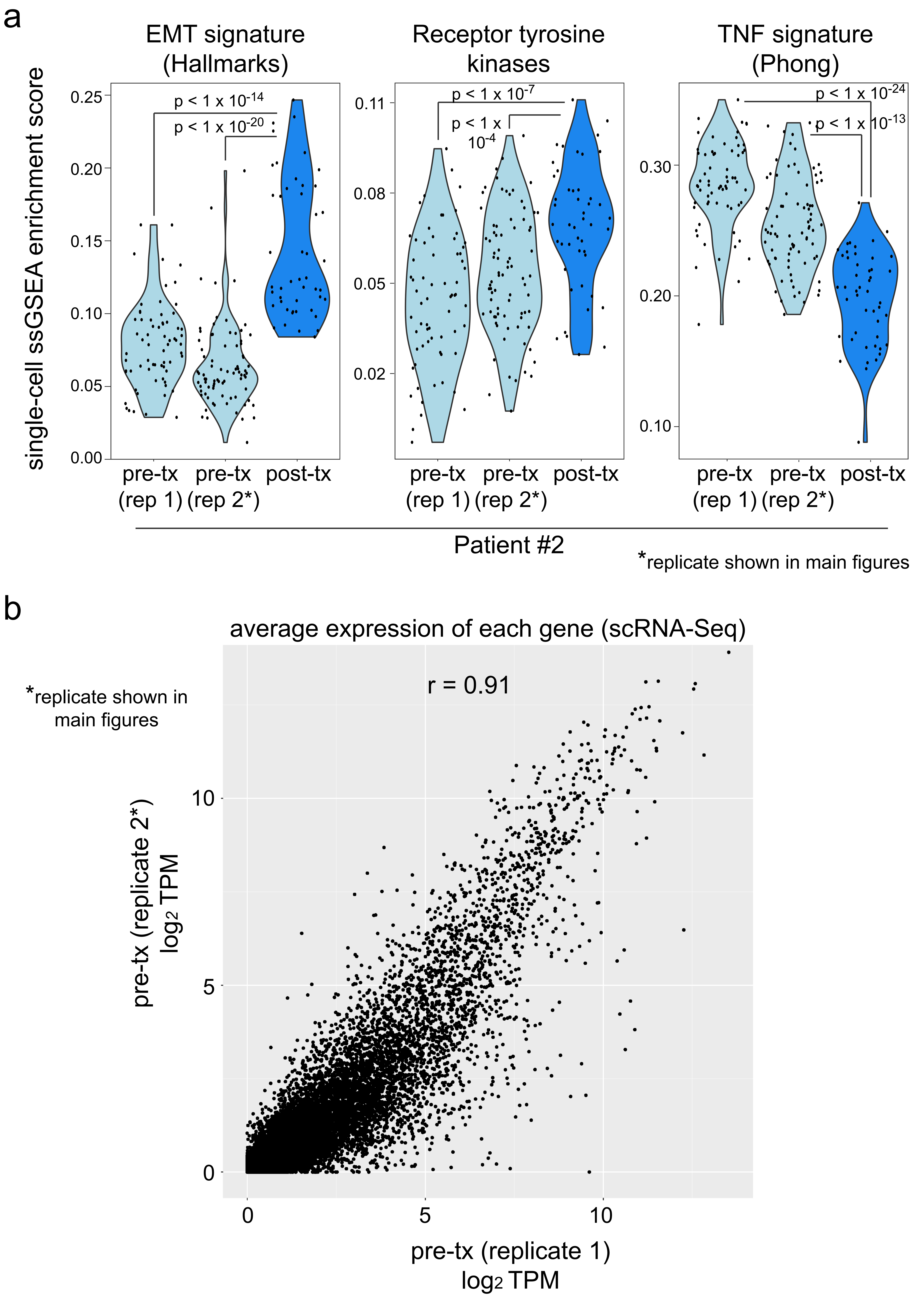

Supplementary Figure 23 | Reproducibility of scRNA-Seq data across different batches. The patient #2 pre-treatment (day 290) specimen was subjected to scRNA-Seq twice on different days using different Fluidigm chips to test batch effects. These two independent runs are referred to as replicate 1 ("rep 1") and replicate 2 ("rep 2"), the latter of which is the replicate shown in the main figures. (**a**) ssGSEA enrichment scores for patient #2 pre-treatment samples (both replicates) and post-treatment for comparison for indicated signatures. Each point represents one cell. p-values are by t-test. (**b**) Average expression of each gene across all single cancer cells (scRNA-Seq) in replicates 1 and 2 of the patient #2 pre-treatment sample. Each point represents the average of one gene in $\log_2(\mathrm{TPM} + 1)$. r value is by Pearson correlation.

Supplementary Figure 24

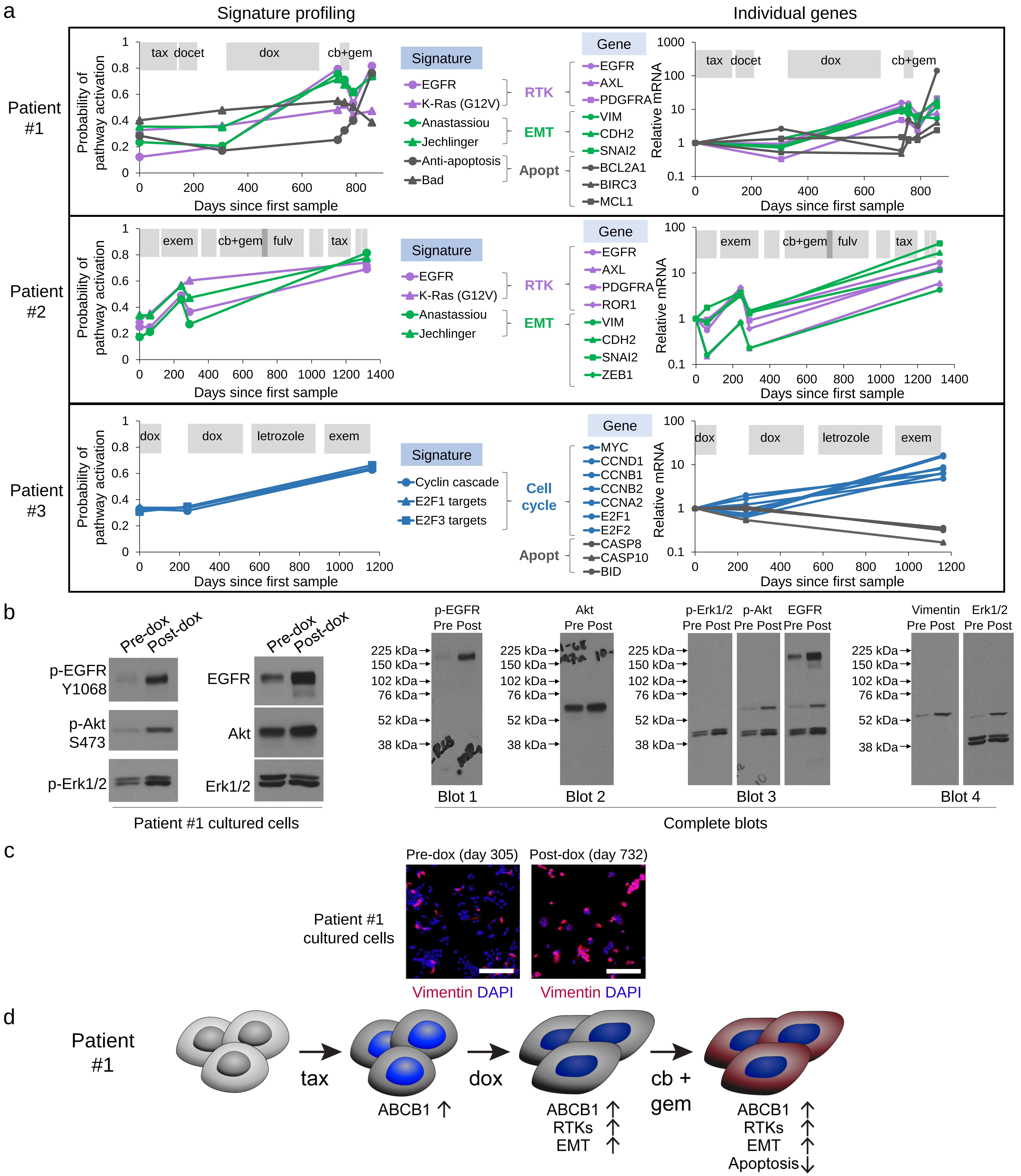

Supplementary Figure 24 | Increased post-treatment EMT, RTK, anti-apoptosis, and proliferation properties ascertained by bulk RNA-Seq. (**a**) ASSIGN signature scores for indicated phenotypes (left) or day 0-normalized gene FPKM gene expression for indicated genes (right) based on bulk RNA-Seq data of longitudinal samples. RTK-associated data are in purple, EMT in green, apoptosis in dark grey, and proliferation in blue. (**b**) Western blot of cultured patient #1 cells (passage 1). Right side shows complete blots; each "Blot" represents a membrane, and blots 3 and 4 were re-probed from left to right. (**c**) Immunofluorescence of cultured patient #1 cells (passage 1). Scale, 200 µm. (**d**) Phenotypic evolution of patient #1. Tax, taxane-based treatment; dox, doxorubicin; cb, carboplatin; gem, gemcitabine.

Supplementary Figure 25

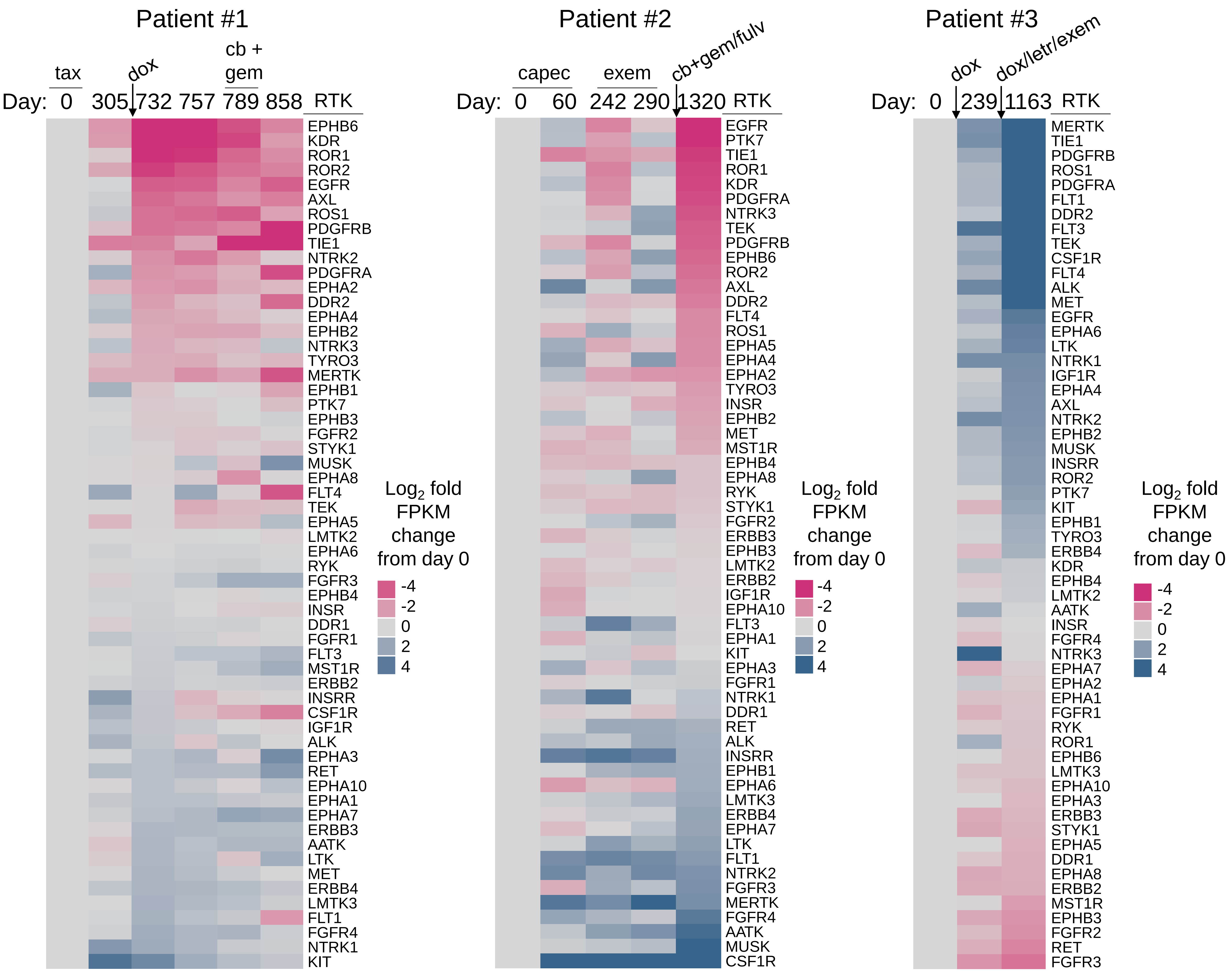

Supplementary Figure 25 | Evolution of RTK gene expression in three breast cancer patients. Heatmaps showing log$_2$ fold change expression from day 0 (FPKM expression values) for all 58 receptor tyrosine kinases over time in breast cancer patients #1 (left), #2 (middle), and right (#3). RTKs are sorted by fold-change expression from day 0 in the post-bottleneck timepoint for each patient.

Supplementary Figure 26

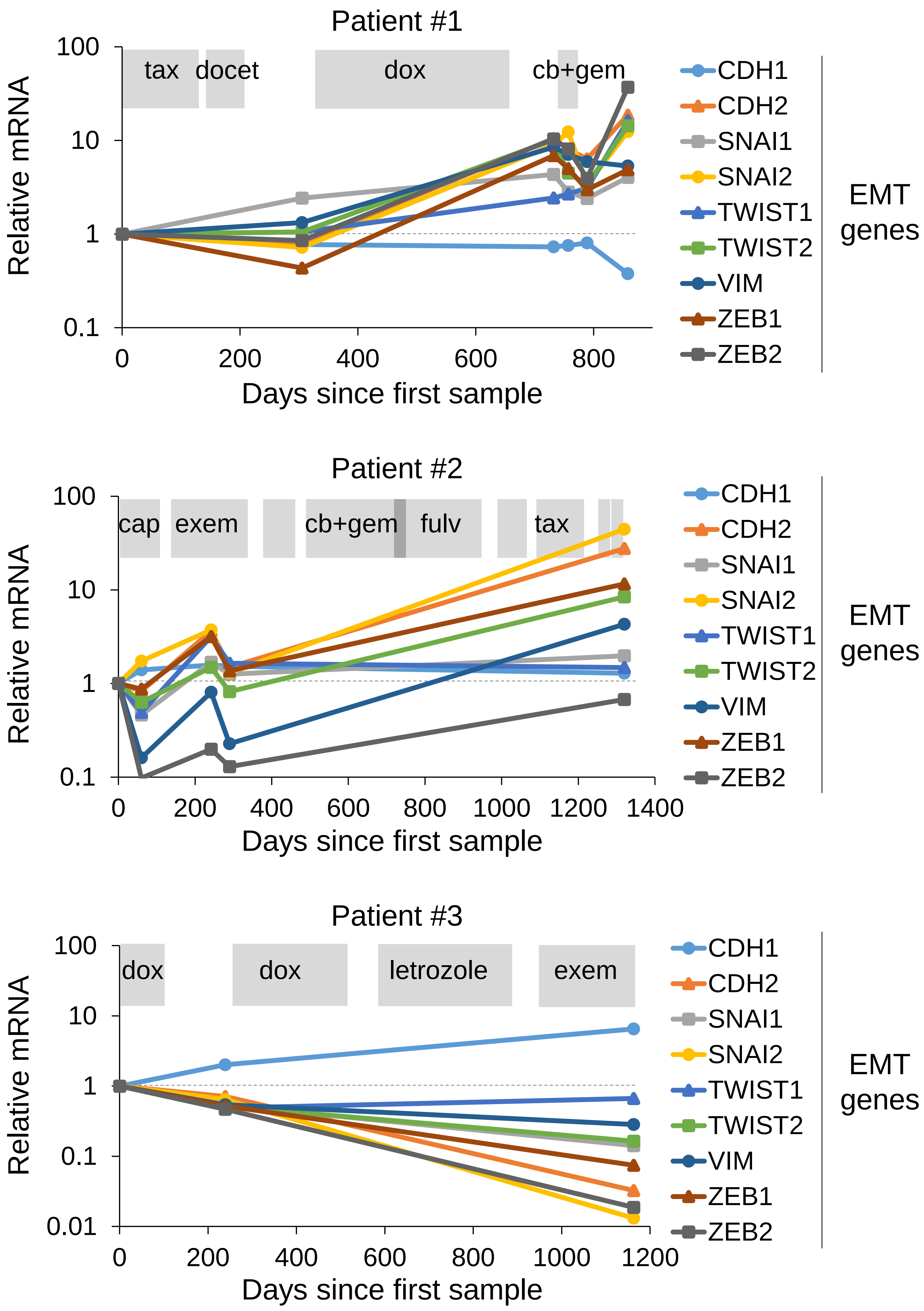

Supplementary Figure 26 | Evolution of EMT-associated gene expression in three breast cancer patients. Day 0-normalized expression (RNA-Seq FPKM values) for EMT-associated transcription factors and other genes for patients #1 (top), #2 (middle), and #3 (bottom) over time with drug treatment indicated in grey. See Fig. 2 legend for drug abbreviation definitions.

Supplementary Figure 27

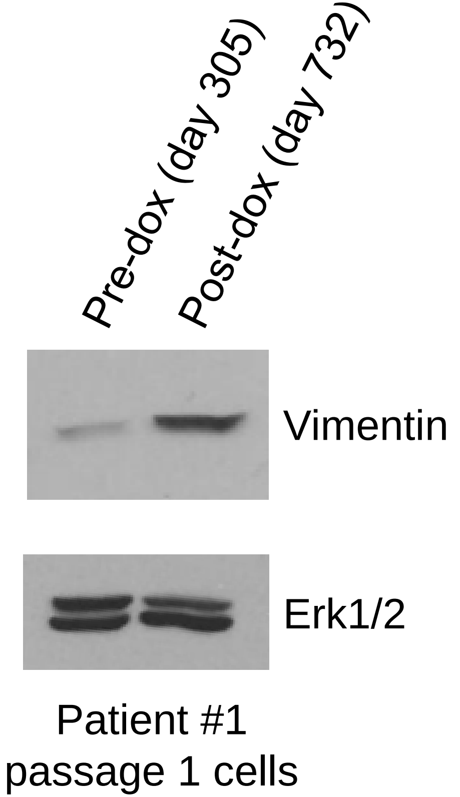

Supplementary Figure 27 | Vimentin is increased in patient #1 cells per Western blot. Western blot of cultured patient #1 pre- (day 305) and post-doxorubicin (day 732) cells using same lysate as in Supplementary Figure 24b and same Erk1/2 loading control. Renaissance medium was used (see Methods). Complete blots are shown in Supplementary Figure 24b.

Supplementary Figure 28

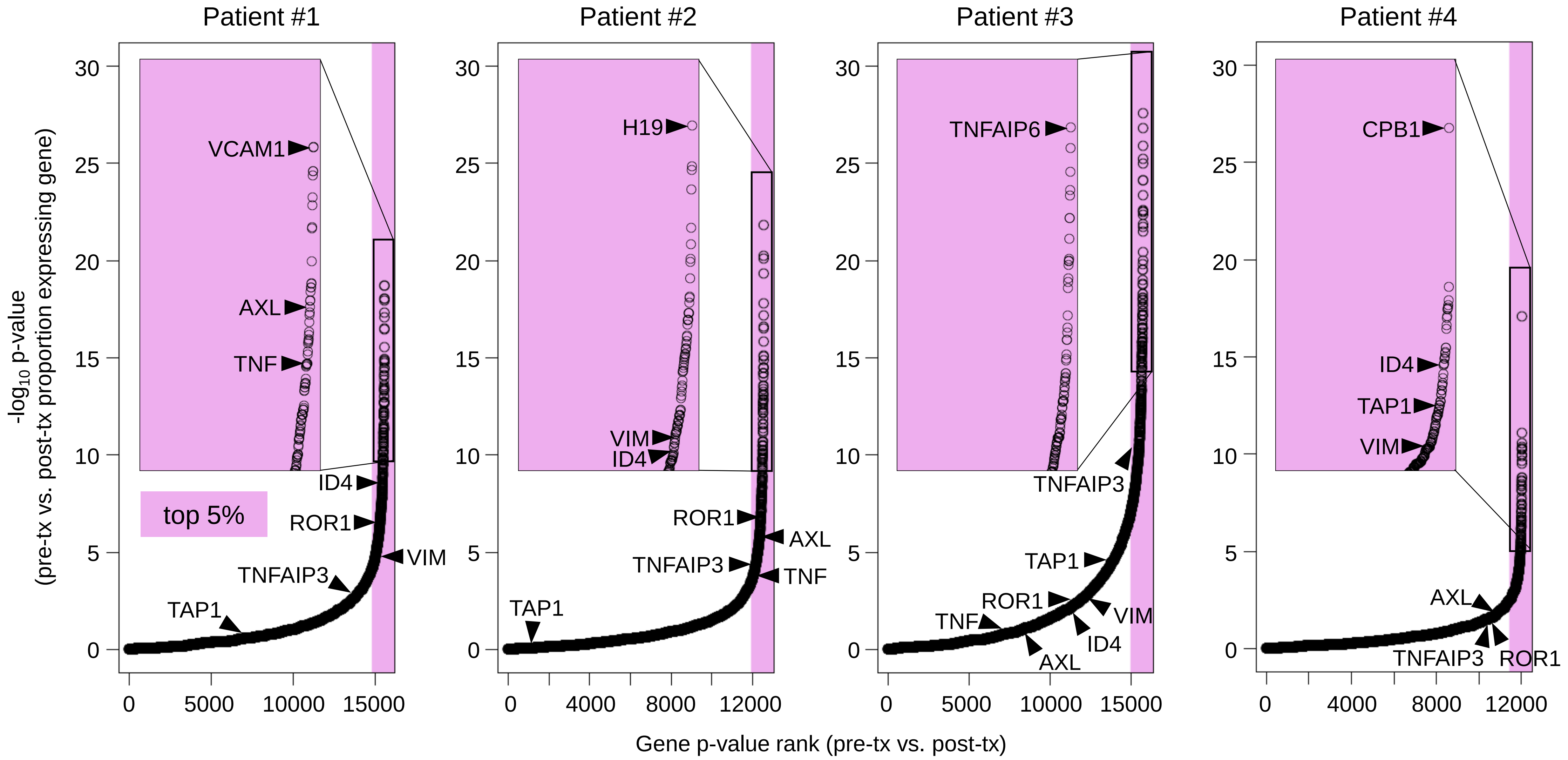

Supplementary Figure 28 | Identification of genes differentially expressed between pre- and post-treatment samples by single-cell RNA-Seq. scRNA-Seq was performed on pre- and post-treatment samples from patients #1-#4 and a two-sample proportion test was performed on each patient for each gene to compare the proportion of cells in pre- vs. post-treatment samples expressing each gene. p-value ranks (x-axis; higher rank indicates higher significances) were plotted against $-\log_{10}$ (p-value) for each gene expressed in at least 1 cell in each patient.

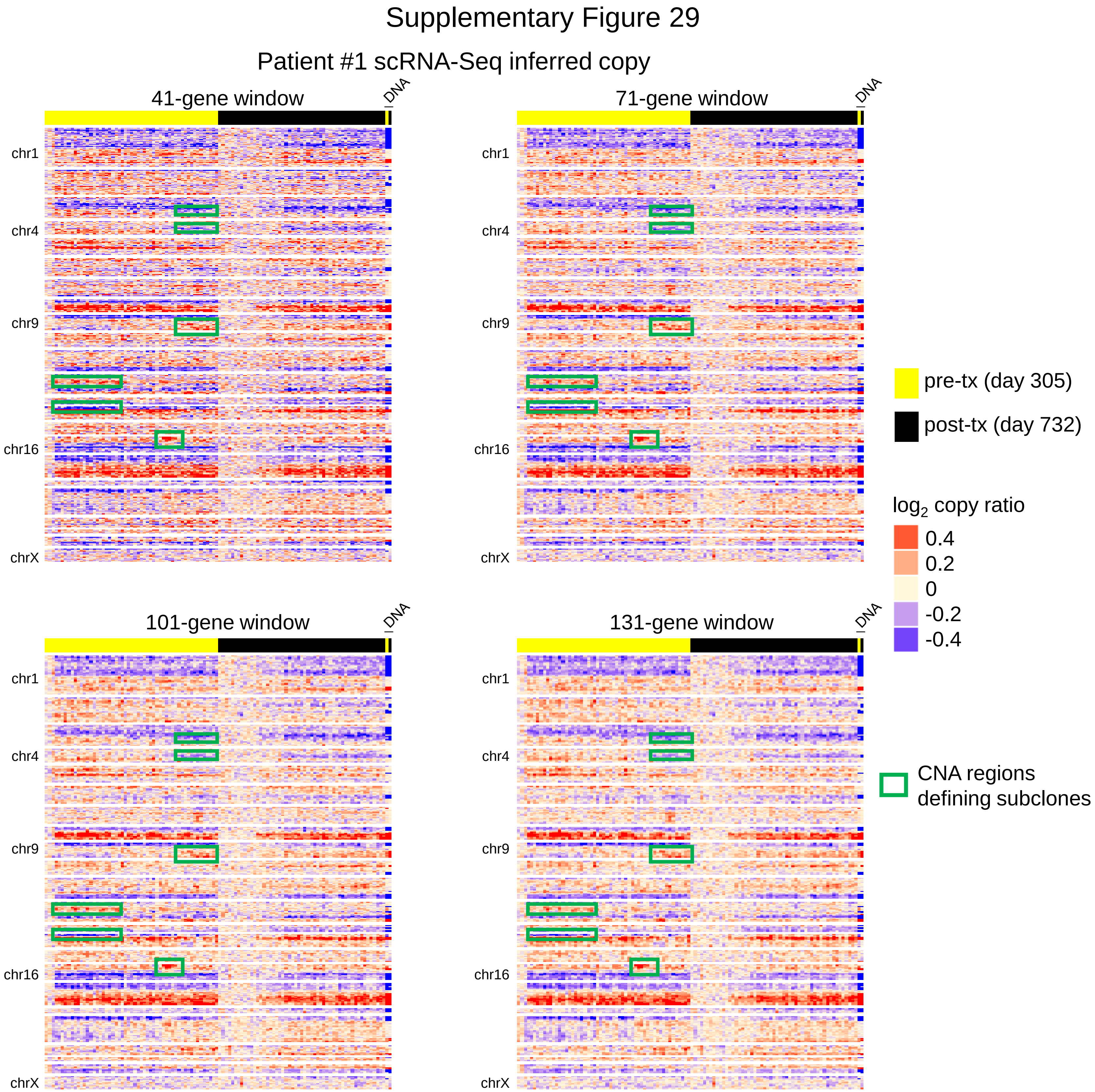

Supplementary Figure 29 | Effects of various window sizes on inferring copy number from single-cell RNA-Seq data. scRNA-Seq was performed on pre- and post-treatment cells from patient #1 and copy number was inferred and corroborated with bulk DNA-Seq-based copy for each gene. The window size for chromosome region gene expression averaging was varied to determine window size effects on inferring CNAs. Each column represents one cell, except where "DNA" is indicated, which indicates CNA inferred from bulk DNA-Seq. Green boxes indicate subclonal CNAs aiding in assignment of cells to subclones.

Supplementary Figure 30

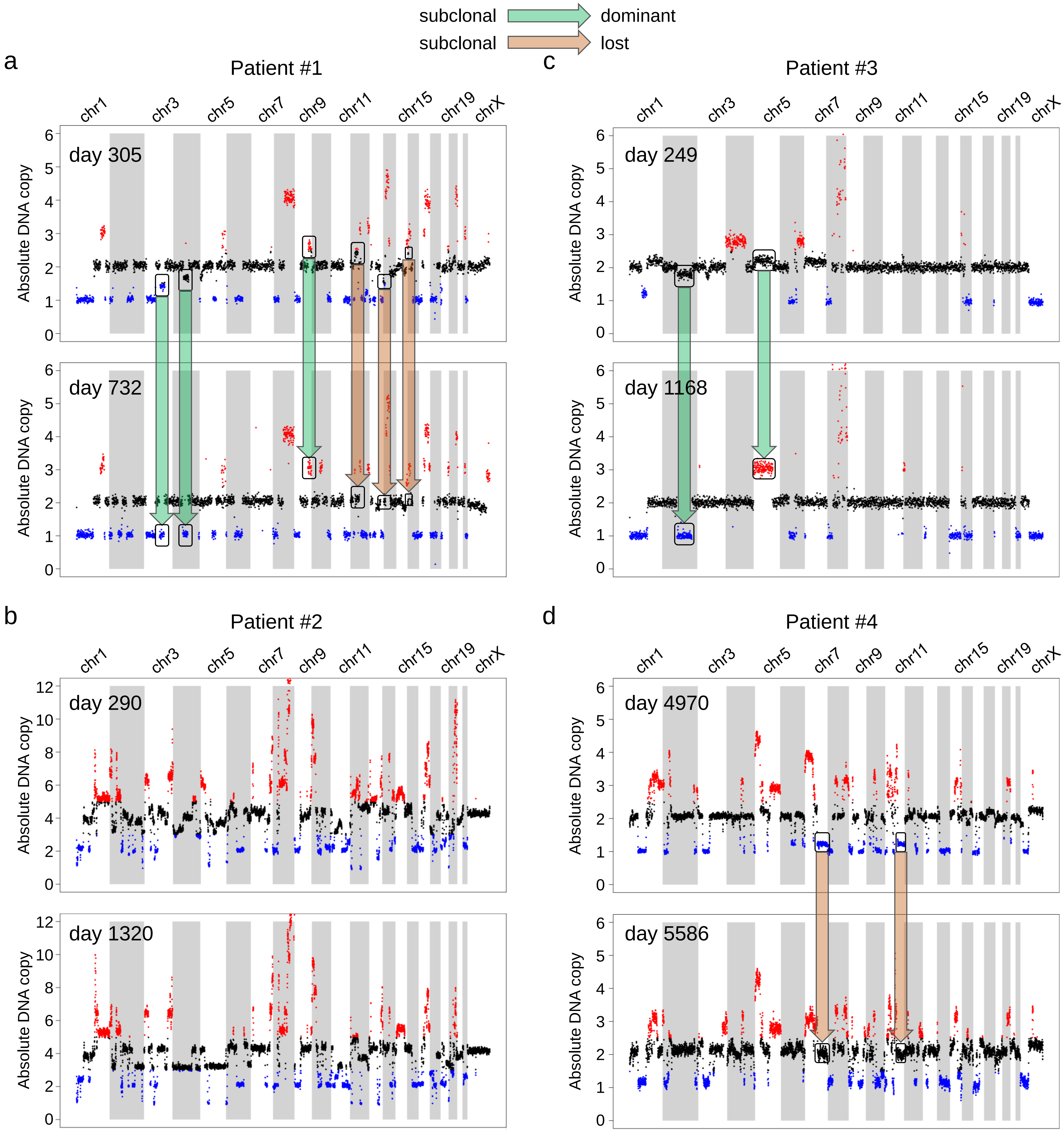

Supplementary Figure 30 | Identification of subclone-specific copy number alterations aiding scRNA-Seq single-cell subclone assignment. Absolute copy of each chromosome region in pre- and post-treatment samples in patients #1-#4 (**a-d**) inferred from WGS. Arrows indicate subclonal CNAs (with absolute copy between integer values) that become dominant (green) or lost (orange) after treatment, and thus define specific surviving or dying subclones. Thus these CNAs can aid in assigning single cells to specific subclones based on their scRNA-Seq copy profile. Red indicates amplification (above 2.5, or 5 for patient #2) while blue indicates deletion (below 1.5, or 3 for patient #2). Patients #1 and #3, 30-segment windows. Other patients, 160-segment.

Supplementary Figure 31

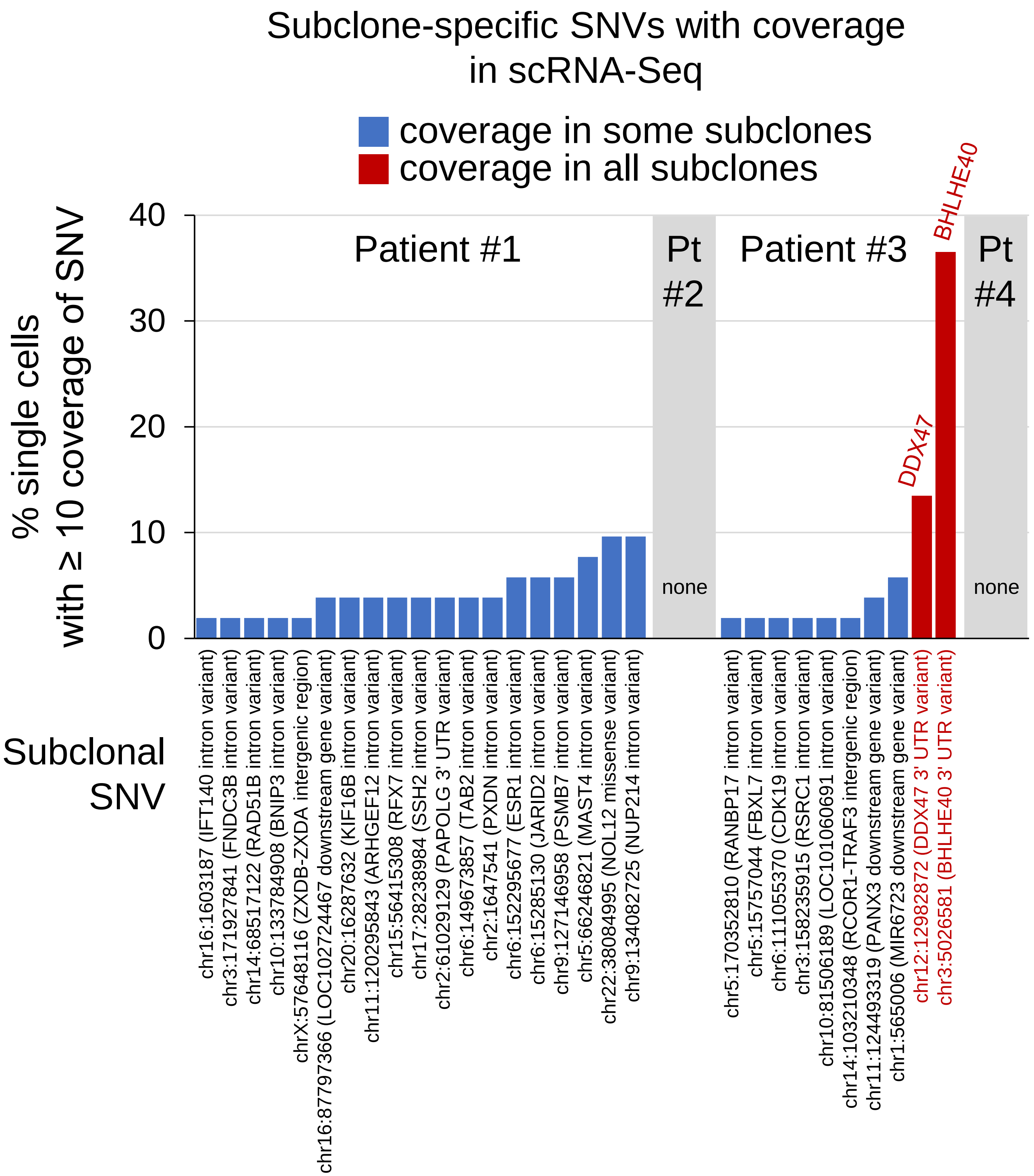

Supplementary Figure 31 | Identification of subclonal SNVs with coverage in scRNA-Seq data. Read depth coverage of sites of subclonal SNVs (non-truncal SNVs defining specific subclones) was determined in patient #1-#4 pre-treatment scRNA-Seq data to identify candidate SNVs for validation of subclone assignment of single cells (determined based on CNA inferred from scRNA-Seq). Y-axis represents the proportion of individual cancer cells assayed by scRNA-Seq with at least 10 read depth at the SNV site regardless of wild-type or mutant status. Only SNVs meeting this criterion in at least one cell were analyzed. Red indicates SNVs that had such coverage in at least one cell from all pre-treatment subclones (greater suitability for verification of subclone assignments), while blue indicates that at least one subclone lacked such coverage in all cells from the subclone (less suitability). Patients #2 and #4 had no SNVs meeting criteria.

Supplementary Figure 32

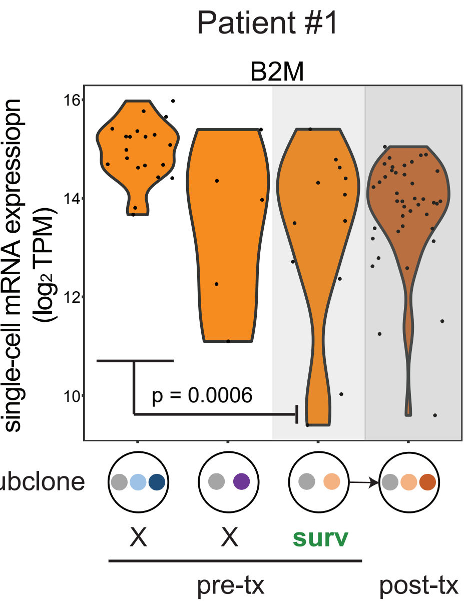

Supplementary Figure 32 | Pre-existence of low antigen presentation phenotype in survivor subclone. *B2M* gene expression in single cells in each patient #1 pre-treatment subclone, along with post-treatment cells. Each dot represents a single cell. p-value is by student's t-test. Subclones correspond to those shown in Fig. 2a. "X" indicates disappearing subclone while "surv" indicates the subclone giving rise to the post-treatment sample.

Supplementary Figure 33

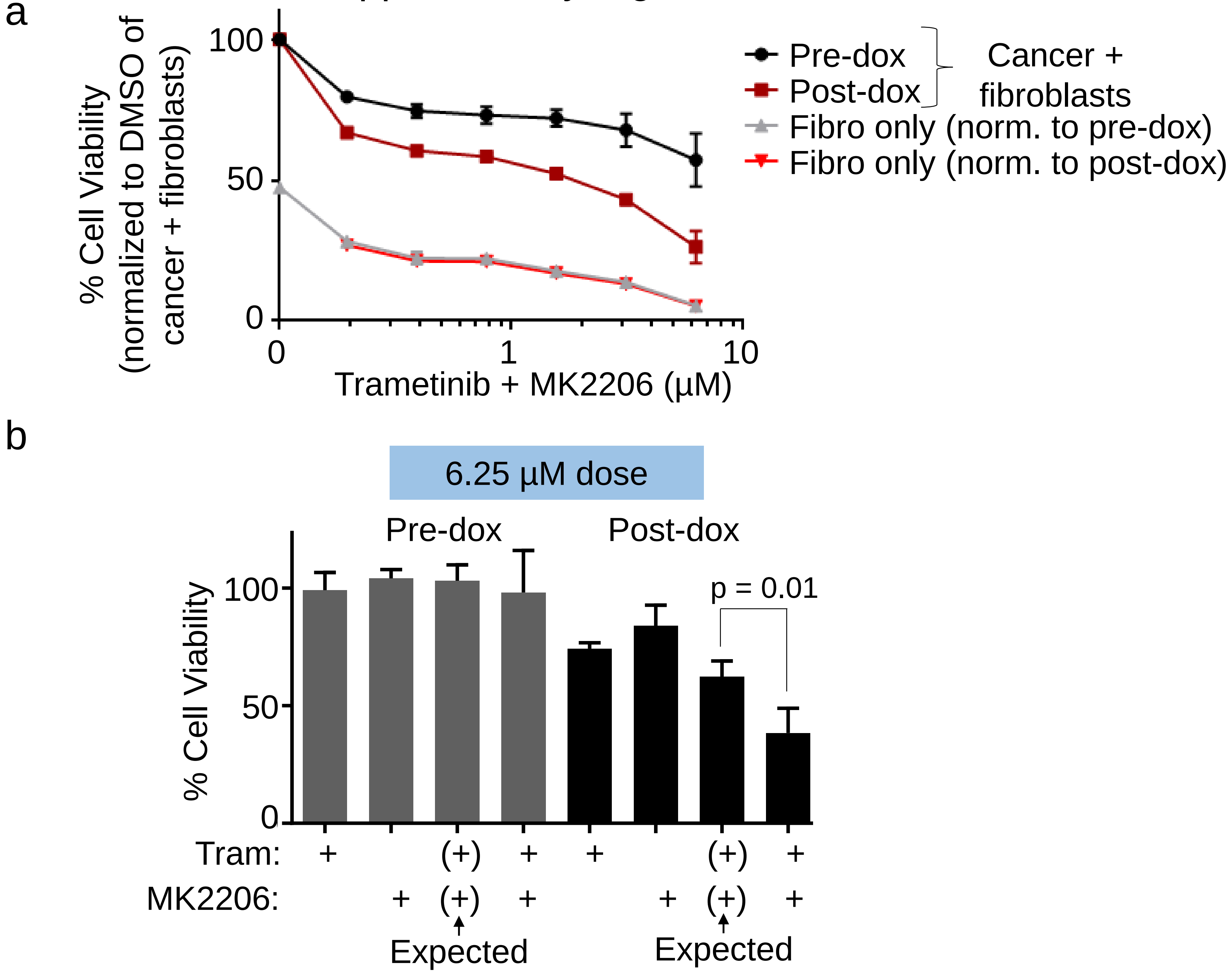

Supplementary Figure 33 | Acquired sensitivity to combined MEK and Akt inhibition in post-doxorubicin patient #1 cells. (**a**) Pre- and post-doxorubicin patient #1 cells grown on fibroblasts (or fibroblasts only with no cancer cells) were treated with equimolar doses of indicated drugs and subjected to CellTiter-Glo assay after 3 days of treatment. Data are normalized to DMSO control mean (= 100%) for cancer + fibroblasts wells. Fibroblast-only wells were normalized to pre-dox (grey triangles) or post-dox (red triangles) cancer + fibroblasts DMSO control mean. Dox, liposomal doxorubicin. Cells were from day 305 (pre-dox) or day 732 (post-dox). (**b**) Synergy analysis of 6.25 µM dose of combined trametinib and MK2206 in pre- and post-doxorubicin cells. Expected is by Bliss independence; p-value is by student's t-test. (Fibroblast-only signal was subtracted from cancer + fibroblast readings.) Error bars indicate s.d. of four technical replicates in both panels.

Supplementary Figure 34

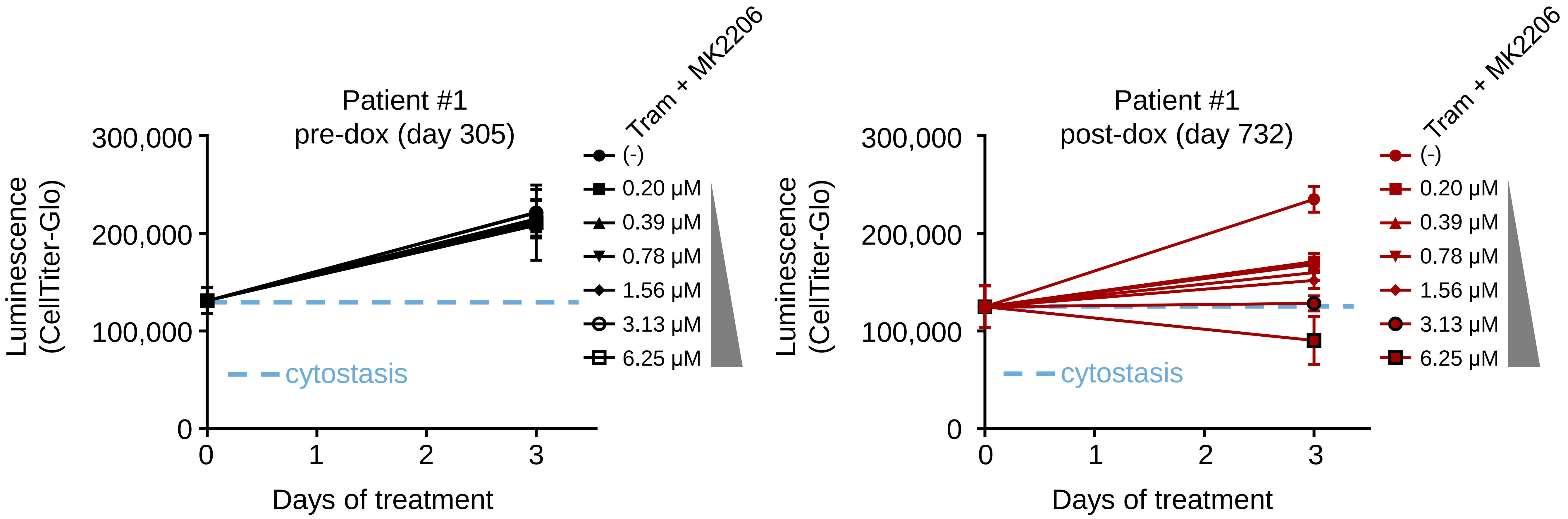

Supplementary Figure 34 | Proliferative capacity of pre- and post-doxorubicin patient #1 cells in response to MEK and Akt co-inhibition. Pre- (left) and post-doxorubicin (right) patient #1 cells were assayed for response to indicated equimolar doses of trametinib and MK2206 combined. These data are from the same assay as shown in Fig. 7a and 7b but also include the CellTiter-Glo luminescence readings taken on day 0 (the day the drug was given). Blue dotted line indicates cytostatic effect, while slopes above this line indicate continued proliferation and slopes below this line indicate decreasing cell number relative to day 0. “(-)” indicates DMSO treatment control. Luminescence readings shown are after subtracting out matched fibroblast-only signal on the same day and same drug treatment to get cancer-only signal. Error bars indicate s.d. of four technical replicates.
