# Supplementary material for: Combating subclonal evolution of resistant cancer phenotypes
Source: Nat Commun. 2017 Nov 1;8:1231. doi: 10.1038/s41467-017-01174-3 (PMC5666005; doi:10.1038/s41467-017-01174-3)
Supplement: Supplementary file 3 — Description of Additional Supplementary Files [file 41467_2017_1174_MOESM3_ESM.pdf]

Description of Additional Supplementary Files

File Name: Supplementary Data 1
Description: Clinical history of breast cancer patient #1

File Name: Supplementary Data 2
Description: Clinical history of breast cancer patient #2

File Name: Supplementary Data 3
Description: Clinical history of breast cancer patient #3

File Name: Supplementary Data 4
Description: Clinical history of breast cancer patient #4
